# Supplementary material for: The hypolipidemic effect of MI-883, the combined CAR agonist/ PXR antagonist, in diet-induced hypercholesterolemia model
Source: Nat Commun. 2025 Feb 6;16:1418. doi: 10.1038/s41467-025-56642-y (PMC11802874; doi:10.1038/s41467-025-56642-y)
Supplement: Supplementary file 1 — Supplementary Information [file 41467_2025_56642_MOESM1_ESM.docx]

**Supplementary Information**

**The hypolipidemic effect of MI-883, the first Combined CAR Agonist/ PXR Antagonist, in Diet-Induced Hypercholesterolemia in Mice**

*Jan Dusek^1^*^,^ *^16^, Ivana Mejdrová^2^*^,^*^16^, Klára Dohnalova^3,4^, Tomas Smutny^1^, Karel Chalupsky^3^, Maria Krutakova^1^, Josef Skoda^1^, Azam Rashidian^5^, Ivona Pavkova^6^, Kryštof Škach^2^,* *Jana Hricová^2^, Michaela Chocholouskova^7^, Lucie Smutna^1^, Rajamanikkam Kamaraj^1^, Miloš Hroch^8^, Martin Leníček^9^, Stanislav Mičuda^10^, Dirk Pijnenburg^11^, Rinie van Beuningen^11^, Michal Holčapek**^7^, Libor Vítek^9,12^, Magnus Ingelman-Sundberg^13^, Oliver Burk^14^, Thales Kronenberger^5,15^, Radim Nencka^2^*, Petr Pavek^1^**

^1^ Department of Pharmacology and Toxicology, Faculty of Pharmacy in Hradec Králové, Charles University, Akademika Heyrovskeho 1203/8, CZ-500 03, Hradec Králové, Czech Republic;

^2^ Institute of Organic Chemistry and Biochemistry, Czech Academy of Sciences, Flemingovo nám. 2, CZ-166 10 Prague 6, Czech Republic;

^3^ Czech Centre for Phenogenomics, Institute of Molecular Genetics of the Czech Academy of Sciences, Vídeňská 1083, CZ-142 20, Prague; Czech Republic;

^4^ First Faculty of Medicine, Charles University, Kateřinská 32, CZ-112 08, Prague, Czech Republic;

^5^ Institute of Pharmacy, Pharmaceutical/Medicinal Chemistry and Tübingen Center for Academic Drug Discovery, Eberhard Karls University Tübingen, Auf der Morgenstelle 8, 72076 Tübingen, Germany;

^6^ Military Faculty of Medicine, University of Defence, Třebešská 1575, Hradec Králové, CZ-500 01, Czech Republic;

^7^ Department of Analytical Chemistry, University of Pardubice, Faculty of Chemical Technology, Studentská 573, CZ-532 10, Pardubice, Czech Republic;

^8^ Department of Biochemistry, Faculty of Medicine in Hradec Králové, Charles University, Šimkova 870, CZ-500 03, Hradec Králové, Czech Republic;

^9^ Institute of Medical Biochemistry and Laboratory Diagnostics, General University Hospital in Prague and First Faculty of Medicine, Charles University, Na Bojišti 3, Praha 2, CZ-120 00, Prague, Czech Republic;

^10^ Institute of Pharmacology, Faculty of Medicine in Hradec Králové, Charles University; Šimkova 870, CZ-500 03, Hradec Králové, Czech Republic;

^11^ PamGene, Wolvenhoek 10, 5211 HH 's-Hertogenbosch, the Netherlands;

^12^4th Department of Internal Medicine, General University Hospital in Prague and First Faculty of Medicine, Charles University, U Nemocnice 2, Praha 2, CZ-128 08, Prague, Czech Republic;

^13^ Section of Pharmacogenetics, Department of Physiology and Pharmacology, Karolinska Institutet, SE-171 77 Stockholm, Sweden;

^14^ Dr. Margarete Fischer-Bosch-Institute of Clinical Pharmacology, Stuttgart, Auerbachstraße 112, 70376 Stuttgart, Germany and University of Tübingen, 72074 Tübingen, Germany;

^15^ School of Pharmacy, Faculty of Health Sciences, University of Eastern Finland, 70211, Kuopio, Finland.

*-corresponding authors

^16^ -these authors contributed equally

**Corresponding Author**

* Professor Petr Pavek, PharmDr., Ph.D. -Department of Pharmacology and Toxicology, Faculty of Pharmacy in Hradec Králové, Charles University, Akademika Heyrovskeho 1203, 500 05, Hradec Králové, Czech Republic; E-mail: [pavek@faf.cuni.cz](mailto:pavek@faf.cuni.cz)

* Radim Nencka, Ph.D. -Institute of Organic Chemistry and Biochemistry, Czech Academy of Sciences, Flemingovo nám. 2, 166 10 Prague 6, Czech Republic; E-mail: [radim.nencka@uochb.cas.cz](mailto:radim.nencka@uochb.cas.cz)

1. **Chemistry**

We harnessed the synthetic approach from our previous study and applied it to a preparation of a small series of fluorinated CAR agonists. Firstly, mono or di- di-fluorinated amino pyridine was reacted with 2-bromo-1-(4-chlorophenyl)ethan-1-one at 70 °C in EtOH leading to 2-substituted imidazo[1,2-*a*]pyridine (**2**) with fluorine atom in the position 7. Next, position 3 was iodinated with NIS in acetonitrile in high yield (**3**). During this fast reaction, a suspension is formed, which allowed a fast work-up of the reaction by filtration. Sonogashira coupling reaction with TMS-acetylene under Pd(PPh_3_)_2_Cl_2_ catalysis provided intermediate **4**. Finally, CuAAC (Huisgen-Meldal) click reaction with 5-(azidomethyl)-2-chlorobenzamide afforded the final compounds **5**.

^a^Reagents and conditions: (a) 2-bromo-1-(4-chlorophenyl)ethan-1-one, NaHCO_3_, EtOH, 70 °C, o.n.; (b) NIS, CH_3_CN 25 °C; (c) TMS-acetylene, CuI, TEA, Pd(PPh_3_)_2_Cl_2_, DMF, 0 °C – 25 °C; (d) 5-(azidomethyl)-2-chlorobenzamide, CuSO_4_.5H_2_O, KF, Na-ascorbate, THF/H_2_O (1:1), 0 °C - rt, 1h.

- 1. **Synthesis of novel ligands**

*General chemical procedures*

NMR spectra were measured on a Bruker Avance II-600 and/or Bruker Avance II-500 instruments (600.1 or 500.0 MHz for ^1^H and 150.9 or 125.7 MHz for ^13^C) in hexadeuterodimethyl sulfoxide, and referenced to the solvent signal (d 2.50 and 39.70, respectively). Mass spectra were measured on an LTQ Orbitrap XL (Thermo Fischer Scientific) using electrospray ionization (ESI) and a GCT Premier (Waters) using EI. The elemental analyses were obtained on a Perkin Elmer CHN Analyzer 2400, Series II Sys (Perkin Elmer), and X-ray fluorescence spectrometer SPECTRO iQ II (SPECTRO Analytical Instruments, Germany). Column chromatography and thin-layer chromatography (TLC) were performed using Silica gel 60 (Fluka) and Silufol Silica gel 60 F_254_ foils (Merck), respectively. Solvents were evaporated at 2 kPa and bath temperature 30 - 60 °C. The compounds were dried at 13 Pa and 50 °C.

*2-(4-Chlorophenyl)-7-fluoroimidazo[1,2-a]pyridine (intermediate compound)* **(2)**

4-fluro-2-aminopyridine **1** was dissolved in EtOH and 2-bromo-1-(4-chlorophenyl)ethan-1-one (1 eq) was added followed by an addition of NaHCO_3_ (1 eq). The reaction mixture was heated at 70 °C overnight. After the completion of the reaction (monitored by TLC or UPLC), the solvent was evaporated to a minimal volume, a residue was diluted with EtOAc and washed with water. The water phase was extracted twice more with EtOAc, and the combined organic phases were dried over sodium sulfate and evaporated. The residue was purified by flash column chromatography providing compound **2**. Mobile phase petrolether/EtOAc (20-70 %). Yield 510 mg (83 %). ^1^H NMR (401 MHz, DMSO-*d*_6_) δ 8.59 (ddd, *J* = 7.5, 5.9, 0.8 Hz, 1H), 8.40 (d, *J* = 0.7 Hz, 1H), 8.01 – 7.89 (m, 2H), 7.51 – 7.46 (m, 2H), 7.43 (ddt, *J* = 10.1, 2.7, 0.8 Hz, 1H), 6.97 (td, *J* = 7.6, 2.6 Hz, 1H). ^13^C NMR (101 MHz, DMSO-*d*_6_) δ 160.04 (d, *J* = 248.4 Hz), 145.15 (d, *J* = 14.3 Hz), 144.32 (d, *J* = 1.5 Hz), 132.72, 132.41, 129.14 (d, *J* = 11.1 Hz), 128.94, 104.74 (d, *J* = 29.4 Hz), 100.36 (d, *J* = 23.7 Hz). HRMS: calculated for [M + H], 247.0438; found, 247.0439.

*2-(4-Chlorophenyl)-7-fluoro-3-iodoimidazo[1,2-a]pyridine (intermediate compound)* **(3)**

Fluorinated 2-(4-chlorophenyl)imidazo[1,2-*a*]pyridine **2** was dissolved in CH_3_CN (5ml/mmol), and NIS (1.05 eq) was added in one portion. The suspension was stirred at 25 °C and the conversion was monitored by TLC. Upon the completion of the reaction (1h), the reaction mixture was evaporated to a minimal volume, afterwards, it was diluted with EtOAc and washed with saturated Na_2_S_2_O_3_ solution. The inorganic phase was extracted twice more with EtOAc, and the combined organic phases were dried over sodium sulfate and evaporated. Alternatively, the suspension was filtered, washed with acetonitrile and the filtrate was evaporated, diluted with EtOAc, and washed with a saturated solution of Na_2_S_2_O_3._ The residue was purified by flash column chromatography, providing compounds **3.** Mobile phase petrolether/EtOAc (10-50 %). Yield: 0.32 g (93 %). ^1^H NMR (401 MHz, DMSO-*d*_6_) δ 8.49 (t, *J* = 6.6 Hz, 1H), 8.07 (d, *J* = 8.2 Hz, 2H), 7.57 (dd, *J* = 9.0, 6.0 Hz, 3H), 7.12 (td, *J* = 7.6, 2.6 Hz, 1H). ^13^C NMR (101 MHz, DMSO-*d*_6_) δ 160.72 (d, *J* = 249.8 Hz), 147.16 (d, *J* = 14.2 Hz), 146.55, 133.05, 132.58, 129.57, 128.94, 128.68, 127.37, 105.83 (d, *J* = 29.7 Hz), 100.73 (d, *J* = 23.8 Hz), 63.43. HRMS: calcd for [M + H], 372.9405; found, 372.9407.

*2-(4-Chlorophenyl)-7-fluoro-3-((trimethylsilyl)ethynyl)imidazo[1,2-a]pyridine* **(4)**

Fluorinated 3-iodoimidazo[1,2-*a*]pyridine **3**  was placed in dried round bottom flask, diluted with dry DMF and degassed at 0 °C and flushed with argon. CuI (10 mol%), Pd(PPh_3_)_2_Cl_2_ (5 mol%) were added and the mixture was properly degassed and dry TEA (3 eq) was added and the mixture degassed again. Finally, TMS-acetylene (5 eq) was added in one portion. The reaction mixture was stirred at 25 °C under the argon atmosphere. After the completion of the reaction (monitored by TLC), the mixture was if necessary diluted with CHCl_3_ and filtered over celite. The filtrate was washed with water, the water phase was extracted twice more with CHCl_3_, and the combined organic phases were dried over sodium sulfate and then evaporated. A residue was purified by flash column chromatography providing compound **4.** Mobile phase petrolether/EtOAc (10-50 %). Yield: 171 mg (58 %). ^1^H NMR (401 MHz, DMSO-*d*_6_) δ 8.44 (dd, *J* = 7.5, 5.6 Hz, 1H), 8.25 – 8.19 (m, 2H), 7.64 – 7.58 (m, 1H), 7.57 – 7.53 (m, 2H), 7.16 (td, *J* = 7.6, 2.6 Hz, 1H), 0.34 (s, 9H). ^13^C NMR (101 MHz, DMSO-*d*_6_) δ 161.38 (d, *J* = 251.3 Hz), 147.05, 145.42, 133.58, 131.78, 128.97, 128.23, 109.11, 106.21 (d, *J* = 29.3 Hz), 101.39 (d, *J* = 24.0 Hz), 92.91, -0.12. HRMS: calcd for [M + H], 343.0834; found, 343.0833.

*2-Chloro-5-((4-(2-(4-chlorophenyl)-7-fluoroimidazo[1,2-a]pyridin-3-yl)-1H-1,2,3-triazol-1-yl)methyl)benzamide* **(5)**

Fluorinated trimethylsilyl(ethynyl)imidazo[1,2-*a*]pyridine derivative **4** was dissolved in THF/H_2_O mixture (1:1) and 5-(azidomethyl)-2-chlorobenzamide (1 eq) was added. The reaction mixture was degassed at 0 °C, refilled with argon and CuSO_4_.5H_2_O (10 mol %), KF (1 eq), Na-ascorbate (1 eq) were added in one portion. The reaction mixture was stirred at 25 °C and monitored by TLC. After the completion of the reaction, the mixture was diluted with EtOAc and washed with water. The water phase was extracted twice more with EtOAc, combined organic phases were dried over sodium sulfate and evaporated. The residue was purified by flash column chromatography providing final compound **5**. Mobile phase petrolether/EtOAc (30-100 %). Yield 75 mg (83 %). ^1^H NMR (401 MHz, DMSO-*d*_6_) δ 8.58 – 8.51 (m, 2H), 7.92 (s, 1H), 7.72 – 7.65 (m, 3H), 7.58 (ddd, *J* = 9.9, 2.7, 0.8 Hz, 1H), 7.53 (s, 1H), 7.49 – 7.38 (m, 4H), 7.05 (td, *J* = 7.6, 2.7 Hz, 1H), 5.75 (s, 2H). ^13^C NMR (101 MHz, DMSO-*d*_6_) δ 167.96, 160.47 (d, *J* = 249.8 Hz), 145.15 (d, *J* = 14.2 Hz), 143.16, 137.68, 136.07, 135.04, 133.02, 132.72, 131.66 (d, *J* = 9.6 Hz), 130.24, 129.63, 129.53, 128.80, 128.32, 127.69 (d, *J* = 11.2 Hz), 125.88, 111.61 (d, *J* = 1.5 Hz), 105.38 (d, *J* = 29.4 Hz), 100.73 (d, *J* = 23.7 Hz), 52.23. HRMS: calcd for [M + H], 481.07412; found, 481.07370.

1. **Plasma protein binding, plasma stability and metabolic stability of MI-883 in liver S9 fractions**

Data for plasma protein binding and plasma stability of MI-883 are presented in Table S1. The elimination constant (k_el_), half-life (t_1/2_), and intrinsic clearance (Cl_int_) were determined^1^ (see Methods, Table S1).

1. **Thallium flux FLIPR-based assay for the identification of hERG potassium channel inhibition**

The test compound MI-883 exhibited no significant inhibition of hERG potassium channel activity and the IC_50_ value could not be defined (Table S1).

**Table S1. Pharmacokinetic parameters of MI-883 in *in vitro* assays.**

|  | **Mouse plasma protein binding** | **Human plasma protein binding** | **Stability in mouse plasma** | **Stability in human plasma** | **Metabolic stability in human liver S9 fraction** | | **Metabolic stability in mouse liver S9 fraction** | | **hERG assay** |
| --- | --- | --- | --- | --- | --- | --- | --- | --- | --- |
|  |  |  | **t1/2, min** | **t1/2, min** | **Clint, μl/min/mg** | **t1/2, min** | **Clint, μl/min/mg** | **t1/2, min** |  |
| MI-883 | 99 % | 99% | >120 | >120 | 2* | 170.8* | 3 | 112.1* | no significant inhibition up to 100 μM |
| CITCO | 98% | n.d | >120 | n.d | 15 | 22.5 | 9 | 39.2 | n.d |
| Midazolam |  |  |  |  | 55±2.5 | 6.3±0.3 | 113 | 3.1 |  |

* estimation, the compound is stable over 60 minutes of the assay


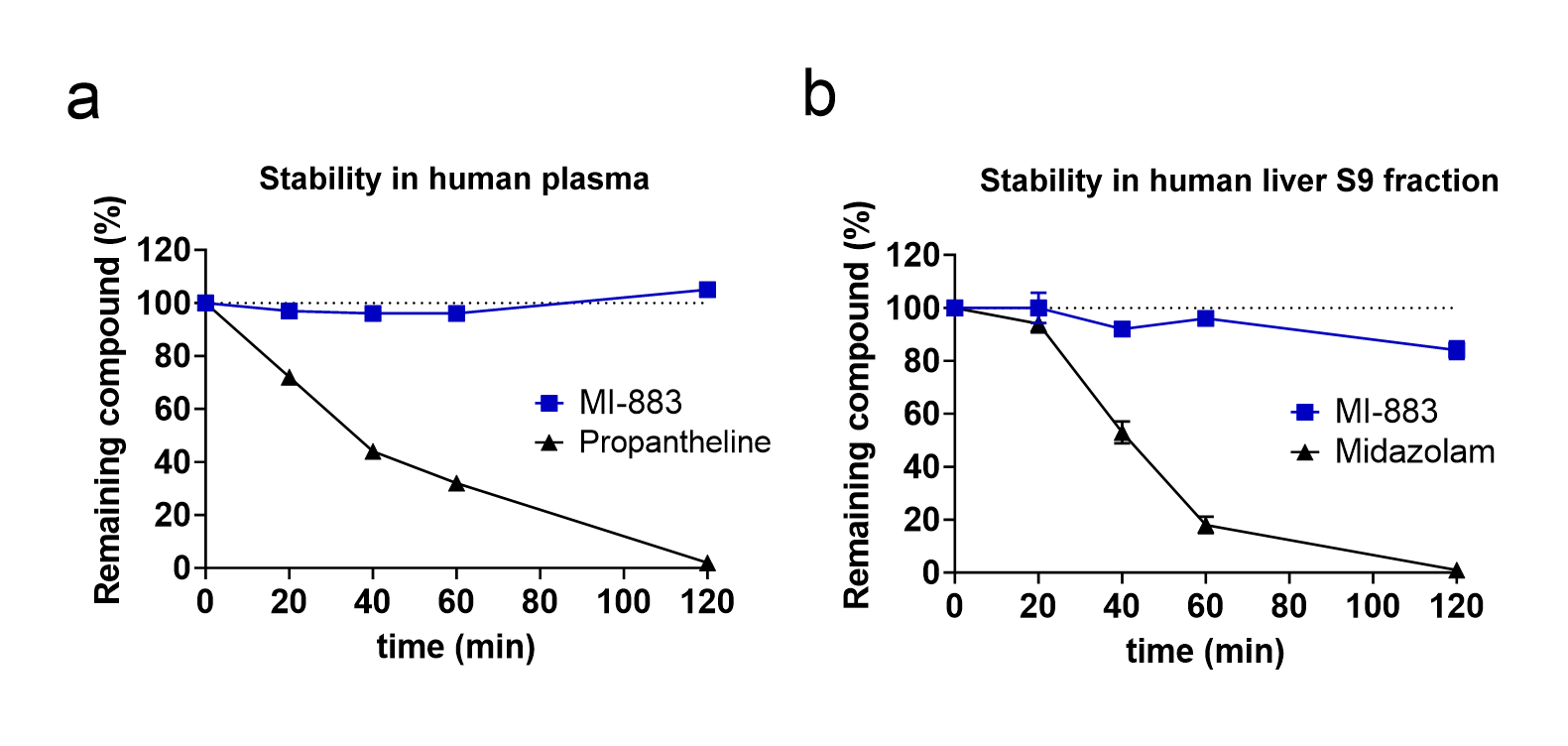


**Supplementary Figure 1.** Metabolic stability of MI-883 in human plasma (**a**) and human liver S9 fraction (**b**). (**a**) Incubation of MI-883 with the polled human serum (from PAA Laboratoria GmbH, Cölbe, Germany) was carried out in 5 aliquots of 70 μL each (one for each time point). Test compounds (1 μM, final DMSO concentration 1%) were incubated at 37 °C with shaking at 100 rpm. Four time points over 120 minutes have been analyzed. The reactions were stopped by adding 420 μL of acetonitrile-water mixture (90:10). Supernatants were analyzed by the HPLC system coupled with a tandem mass spectrometer. The percentage (in % of initial concertation) of the test compounds remaining after incubation in plasma and their half-lives (t1/2) were calculated. (**b**) Metabolic stability of compound MI-883 was assessed at a single concentration (2 μM) at t = 0, 20, 40, 60 and 120 min intervals. The stability of the compound was tested in human liver S9 fraction. Cofactors were used such as UDPGA (for glucuronidation), PAPS (for sulfation), and reduced glutathione (for conjugation of GSH). Compounds were tested in triplicate with or without NADPH. Propantheline and midazolam were used as unstable control compounds in the experiments. Data represent the means±S.D. from at least three independent samples.

1. **Vectors for luciferase reporter gene assays for human PXR, CAR, mouse Car, and CAR3**

Expression vectors (based on pcDNA3.1+/C-(K)-DYK vector) for CAR variant 3 (CAR3, 353 AA, CloneID OHu34914, XM_005245697.4, transcript variant X4, mRNA) and CAR wild type (wtCAR, 348 AA, Clone ID OHu09315, NM_005122.4, transcript variant 3) were purchased from Genscript (Piscataway, NJ, USA). The mouse Car expression vector pCMV6-mCar (NM_009803) was obtained from OriGene Technologies, Rockville, MD, USA).

The CYP3A4 promoter luciferase reporter construct with a distal XREM (−7836/-7208) and a basal promoter sequence (prPXRE, −362/+53) from the CYP3A4 gene promoter region (CYP3A4-luc) and expression vector for human PXR (Clone ID:OHu23779D, OHu23779D_pcDNA3.1+/C-(K)-DYK, NM_003889.3) were used for the PXR-dependent luciferase reporter gene assays (Fig. 1i, done at Charles University).

CYP3A4 enhancer/promoter reporter gene plasmid pGL4-CYP3A4(-7830Δ7208-364) used at Dr. Margarete Fischer-Bosch-Institute of Clinical Pharmacology was described before^2^. Expression plasmids encoding human wild-type PXR LBD (wtPXR LBD)^3^, LBP-filled triple mutant PXR(S208W/S247W/C284W)^2^, hereinafter referred to as mutant PXR LBD, and human RXRα^4^ have been described previously. pCYP3A4(ER6)-luc construct contains three copies of the CYP3A4 proximal promoter ER6 response element cloned into the NheI- and BglII-digested site of the pGL4.23 vector containing a minimal promoter (Promega).

- 1. **MI-883 inhibits PXR activation in stably transfected H-P cells**

We also analyzed PXR-dependent CYP3A4 reporter gene assays in H-P cells, which represent HepG2 cells stably over-expressing human PXR, due to transfection of PXR expression plasmid pcDhPXR^5^. Transient batch transfection was performed as described above in section 5.2., using 0.3 µg pGL4-CYP3A4(-7830Δ7208-364) per well. 0.01 µg *Metridia* luciferase expression plasmid pMetLuc2control per well was included. Treatment with chemicals, cell lysis, and measurement of firefly and Metridia luciferase activities was done as described above in the Methods section.

We observed a dose-dependent inhibition of the rifampicin-induced activation of CYP3A4 reporter activity by MI-883 (Supplementary Fig. 2).


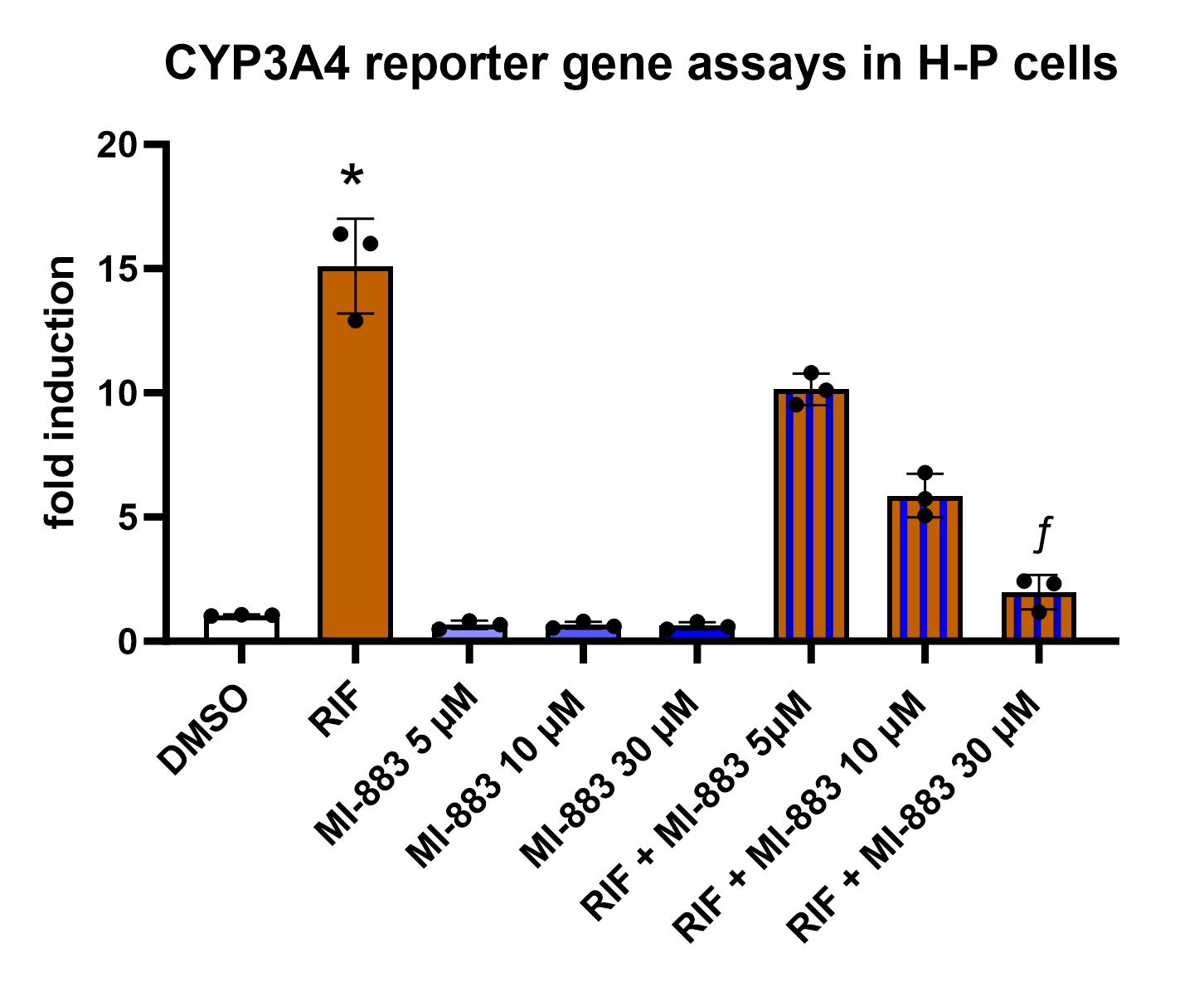


**Supplementary Figure 2.** PXR-dependent CYP3A4 reporter gene assays in H-P cells. MI-883 inhibits PXR activation with rifampicin (10 μM) in a dose-dependent manner. ^*^*P*<0.05, statistically significant activation *vs.* control; ^ƒ^*P*<0.05, statistically significant suppression of PXR activity by MI-883 *vs.* rifampicin (n=3). Data represent means± S.D. (ANOVA with multiple comparisons, n≥3).

1. **Pharmacokinetic study after single-dose application**

**5.1. Reagents and equipment for HPLC-MS/MS analysis**

The following reagents were used: DMSO Chromasolv Plus, HPLC grade, ≥99.7% (Sigma-Aldrich, USA; Cat #34869), Acetonitrile Chromasolv, gradient grade, for HPLC, ≥99.9% (Sigma-Aldrich, USA; Cat #34851), Methanol Chromasolv Plus, for HPLC, ≥99.9% (Sigma-Aldrich, USA; Cat 34860), Formic acid for mass spectrometry, ~98% (Fluka, USA; Cat #94318), BD Microtainer® Blood Collection Tubes, K2EDTA, 0.5 ml (BD, USA), and 2,2,2-Tribromoethanol 97% (Sigma-Aldrich; Cat # T48402). Compound IS-19851 was used as an internal standard (IS). The formulation (2 mg/ml) with the DMSO – PEG300 – Corn oil (5%:5%:90%) was used.

The final mixture was vortexed for 2 min. The compound was fully dissolved in the formulation vehicle. Gradient HPLC system (Shimadzu, Japan) and MS/MS detector API 3000 PE with TurboIonSpray Electrospray module (PE Sciex, USA) were used. VWR Membrane Nitrogen Generators N2-04-L1466, nitrogen purity 99%+ (VWR, USA) was used to generate nitrogen. The Bullet Blender®, model BB24-AU (Next Advance, USA) was used to prepare samples.

- 1. **HPLC-MS/MS conditions**

***Chromatographic conditions:***

Column: Discovery HS C18 (50 x 2.1 mm, 5μm)

Mobile phase А: Acetonitrile: Water : Formic acid = 50 : 950 : 1

Mobile phase B: Acetonitrile: Formic acid = 100 : 0.1

Linear gradient: 0 min 10% B, 1.00 min 100% B, 1.10 min 100% B, 1.11 min 10% B, 2.30 min stop

Elution rate: 400 μL/min. A divert valve directed the flow to the detector from 1.3 to 1.9 min

Column temperature: 30°C

***MS/MS detection:***

Scan type: Positive MRM, Ion source: Turbo spray, Ionization mode: ESI

Nebulize gas: 15 L/min, Curtain gas: 8 L/min, Collision gas: 4 L/min

Ionspray voltage: 5000 V, Temperature: 400°C

| **Other MS parameters** Compound ID | Parent, m/z | Daughter, m/z | Time, ms | DP, V | FP, V | EP, V | CE, V | CXP, V |
| --- | --- | --- | --- | --- | --- | --- | --- | --- |
| **MI-883** | 480.990 | 285.0 | 40 | 56 | 370 | 11 | 39 | 48 |
| IS-19851 | 390.063 | 354.1 | 40 | 71 | 370 |  | 51 | 18 |
| 2-chloro-5-((4-(2-(4-chlorophenyl)-7-fluoroimidazo[1,2-*a*]pyridin-3-yl)-1*H*-1,2,3-triazole-1-yl)methyl)-*N*-methylbenzamide | 495.1 | 495.1 | 15 | 56 | 370 | 11 | 5 | 30 |
| 2-chloro-5-((4-(2-(4-chlorophenyl)-7-fluoroimidazo[1,2-*a*]pyridin-3-yl)-1*H*-1,2,3-triazol-1-yl)methyl)-*N,N*-dimethylbenzamide | 509.1 | 509.1 | 15 | 56 | 370 | 11 | 5 | 30 |
| 2-(4-chlorophenyl)-7-fluoro-3-(1*H*-1,2,3-triazol-4-yl)imidazo[1,2-*a*]pyridine | 314.1 | 314.1 | 15 | 56 | 370 | 11 | 5 | 30 |

Calibration standards for quantification of MI-883 in plasma samples, in the liver, kidney, muscle (Leg triceps), colon, brain, adipose tissue samples and small intestine samples were done with a series of calibration solutions with final concentrations of 5 000, 2 500, 1 000, 500, 250, 100, 50, 25, 10, 5 and 2 ng/ml. Similarly, calibration standards for quantification of MI-883 in urine, feces, and bile samples were done. Correlation coefficients were higher than 0.999. Preparation of calibration curves and calibration curve data are available after request.

- 1. **Pharmacokinetic parameters of MI-883 (10 mg/kg) in C57BL/6N mice after a single dose application**

Pharmacokinetic parameters of MI-883 in C57BL/6N mice after peroral (gavage) and *i.p.* application of a single dose (10 mg/kg) have been calculated as described previously ^1^. No expected metabolites

2-chloro-5-((4-(2-(4-chlorophenyl)-7-fluoroimidazo[1,2-*a*]pyridin-3-yl)-1*H*-1,2,3-triazol-1-yl)methyl)-*N*-methylbenzamide, 2-chloro-5-((4-(2-(4-chlorophenyl)-7-fluoroimidazo[1,2-*a*]pyridin-3-yl)-1*H*-1,2,3-triazol-1-yl)methyl)-*N*-dimethylbenzamide or 2-(4-chlorophenyl)-7-fluoro-3-(1*H*-1,2,3-triazol-4-yl)imidazo[1,2-*a*]pyridine were detected in plasma and tissues.

**Table S2. Pharmacokinetic parameters of compound MI-883 in C57BL/6N mice following intraperitoneal and peroral** **administration (10 mg/kg)**

| **Sample** | **Administration** | **T_max_**  **(min)** | **C_max_ (ng/mL)** | **T_1/2_** | **Peroral bioavailability (%, *p.o.* *vs*. *i.p.)*** |
| --- | --- | --- | --- | --- | --- |
| **Plasma** | *i.p.* | 30 | 5 530 | 217 | 98.6* |
|  | *p.o.* | 30 | 9 290 | 170 |  |
| **Liver** | *i.p.* | 30 | 14 100 | 152 |  |
|  | *p.o.* | 30 | 14 900 | 211 |  |

*estimation of peroral bioavailability based on comparison of AUCs after *p.o*. and *i.p.* application

1. **CYP enzymatic activity assays**

CYP450-Glo™ CYP3A4 and CYP450-Glo™ CYP2B6 Assays (Promega, Hercules, CA) were used to evaluate interaction of MI-883 with these enzymes *in vitro*. The data are presented in Supplementary Fig. 3d, e.


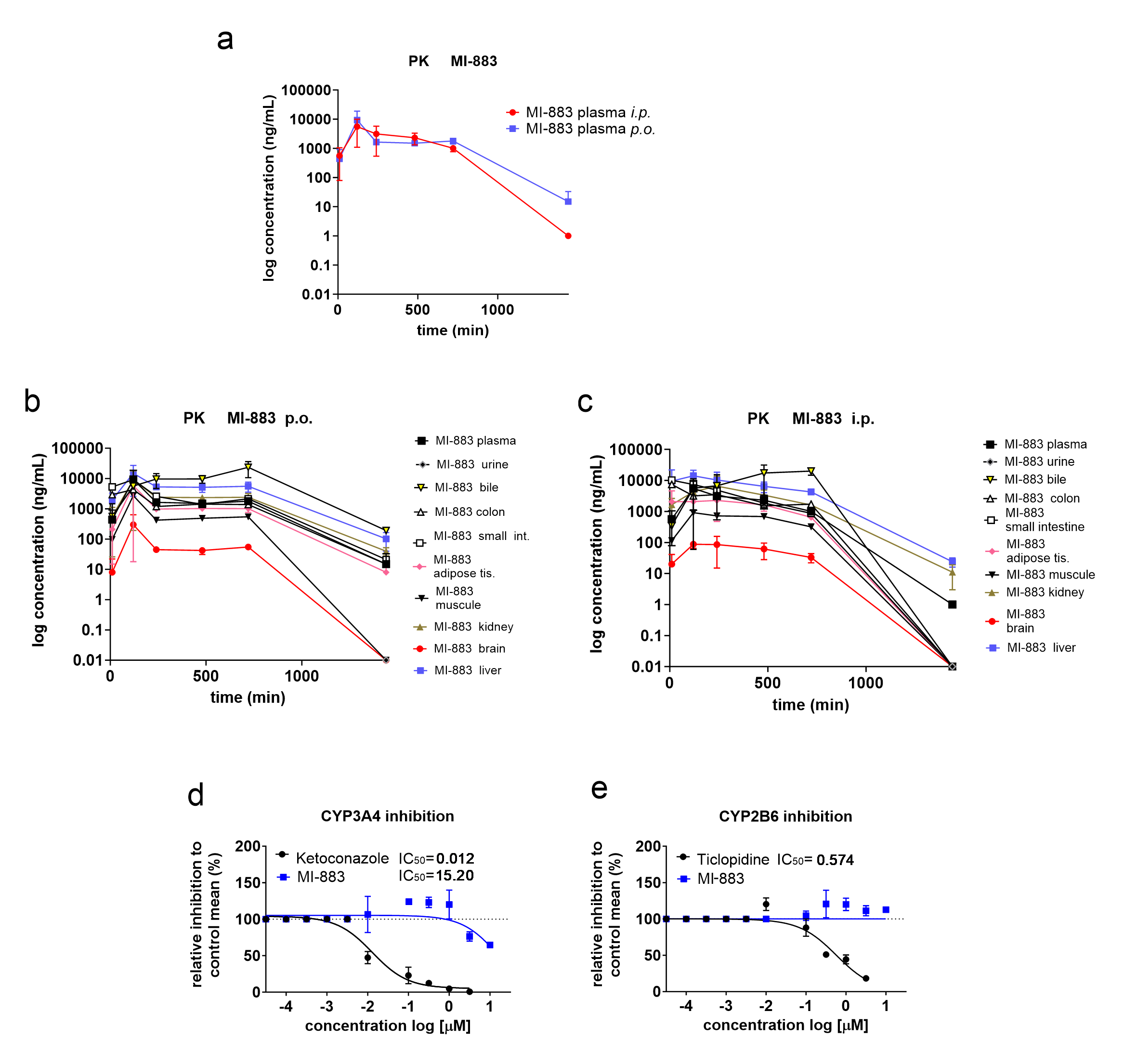


**Supplementary Figure 3.** **Pharmacokinetic characterization of MI-883.** Pharmacokinetic profiles and tissue distribution of MI-883 after *p.o.* (**a**,**b**) and *i.p.* (**a**, **c**) application (10 mg/kg) in male C57BL/6N mice (*n*=4 for each point, mean ±S.D.). Plasma, bile, urine, and tissue samples have been collected and prepared and HPLC/MS/MS analyses have been performed as described in Methods section.

Inhibition of human CYP3A4 (**d**) and CYP2B6 (**e**) enzymatic activities by MI-883 or prototype inhibitors ketoconazole or ticlopidine, respectively, in CYP450-Glo CYP3A4 Assay and CYP2B6 Assays (Promega). Data are presented as means±S.D. from three independent measurements.

1. **Interaction of MI-883 with selected nuclear receptors in cellular luciferase reporter gene assays.**

Gal4-LBD constructs of tested nuclear receptors have been used together with pGL4.31[luc2P/GAL4UAS/Hygro] luciferase reporter construct and pRL-TK *Renilla* expression vector (both from Promega, Hercules, USA) in HepG2 cells transfected with Lipofectamine 2000 or 3000 reagents. For GRα and FXR receptors, full-length expression vectors have been used with pGRE-luc or pFXRE-luc luciferase constructs. For the aryl hydrocarbon receptor (AhR), p1A1-luc construct was used with only pRL-TK vector for transfection normalization. A dual reporter assay (Promega, Hercules, USA) was used to analyze both firefly and *Renilla* luciferase activities. MI-883 and CITCO have been tested at 10 μM concentration. The protocols are described in our recent paper^1^. Prototype ligands were used in the following concentrations: 10 μM GW3965 (LXRα and LXRβ agonist), 10 μM rosiglitazone, 10 μM fenofibrate, 10 μM GW501516, 10 μM thyroxin, 10 μM 6α-ethyl-chenodeoxycholic acid (6-ECDCA), 0.1 μM dexamethasone, 10 μM 3-methylcholanthrene (an agonist of the AhR transcription factor), 10 μM estradiol, 100 nM calcitriol, and mouse Car ligand TCPOBOP (10 μM).


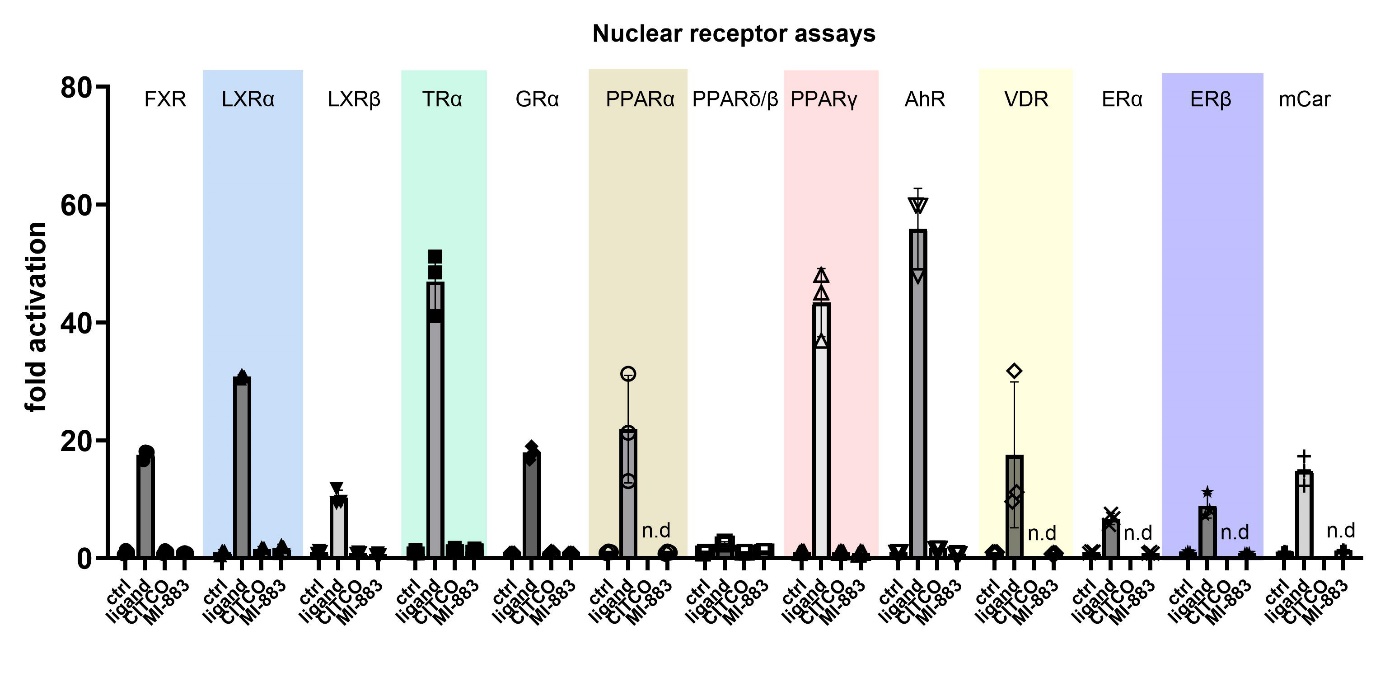


**Supplementary Figure 4.** Interaction of MI-883 with selected nuclear receptors in cellular luciferase reporter gene assays. Gal4-LBD constructs of tested nuclear receptors have been used together with pGL5-luc luciferase reporter construct and pRL-TK Renilla expression vector in HepG2 cells transfected with Lipofectamine 2000 or 3000 reagents. For GRα and FXR receptors, full-length expression vectors have been used with pGRE-luc or pFXRE-luc luciferase constructs. For the aryl hydrocarbon receptor (AhR), p1A1-luc construct was used with only pRL-TK vector for transfection normalization. A dual reporter assay (Promega, Hercules, USA) was used to analyze both firefly and *Renilla* luciferase activities after 24 hours of treatment (*n*=3). MI-883 and CITCO have been tested at 10 μM concentration. Prototype ligands were used in the following concentrations: 10 μM GW3965 (LXRα and LXRβ agonist), 10 μM rosiglitazone, 10 μM fenofibrate, 10 μM GW501516, 10 μM thyroxin, 10 μM 6-ethylchenodeoxycholic acid (6-ECDCA), 0.1 μM dexamethasone, 10 μM 3-methylcholanthrene (an agonist of the AhR transcription factor), 10 μM estradiol, 100 nM calcitriol, and mouse Car ligand TCPOBOP (10 μM). Data are presented as a fold activation to control (vehicle DMSO 0.1%-treated) cells. n.d. -not determined for CITCO. Bars represent means±S.D. (n=3).

1. **Molecular dynamic simulation of human CAR and PXR**

**8.1. MI-883 stabilizes wtCAR into an agonist-bound geometry**

We used an *in silico* approach to inspect the ligand-induced changes in the wild-type CAR ligand binding domain’s (wtCAR-LBD) conformational dynamics. We generated a model for wtCAR/MI-883’s potential binding mode using docking in human wtCAR/CITCO structure (PDB ID: 1XVP)^6^. This model underwent 4 μs of all-atom MD simulations, which were compared against similar simulations with wtCAR/CITCO^1^. The RMSD computed from the trajectory suggests that the simulation has equilibrated and converged around ~2 Å at the end of the simulation (Supplementary Fig. 5) and that the ligands are stable inside the ligand binding pocket, as seen by the ligand’s RMSD (Supplementary Fig. 6). Meanwhile, the overall dynamic behavior of proteins demonstrated by root-mean-square fluctuations (RMSF) displayed a similar pattern of residue fluctuations over the course of the simulation (Supplementary Fig. 7). Trajectory visualization revealed a U-shaped conformation of MI-883 within the wtCAR-LBD, similar to CITCO (6-(4-chlorophenyl)imidazo[2,1-*b*][1,3]thiazole-5-carbaldehyde-O-(3,4-dichlorobenzyl)oxime), the prototype human CAR ligand (Fig. 2a,b). Protein–ligand interactions revealed a similar hydrophobic interaction profile for both ligands, particularly with residues F161 and Y224 (~100% of the analyzed trajectory) and H203 imidazole ring (~85-100% of the analyzed simulation time, Fig. 2c). Other residues including C202, F234, L242, and Y326 also play a minor role in stabilizing both CITCO and MI-883 within the CAR-LBP (Fig. 2a, b). Some interactions, however, are compound-specific such as I164, which is only observed with MI-883 (Fig. 2c). Concurrently, studying the polar interactions revealed the relevant role of H203 in stabilizing both ligands (~50%, Fig. 2d). This interaction occurs with the MI-883’s 7-fluoroimidazo[1,2-*a*]pyridine core and imidazothiazole in CITCO structure. In addition, MI-883 amide group interacts with T225 and D228 backbone oxygen atoms (located on H6) roughly 30%. These polar contacts are absent in the CITCO binding mode, which relies only on the interaction with H203 (~50%) (Fig. 2c, d).

It is known that the closeness of H12 to H3 in wtCAR is the reason for its constitutive activity^6^. To explore the effect of MI-883 on this region, we calculated the distance between H12 and H3 (the center of mass of each helix) along the simulation trajectory. The values for both systems are in the same range (~12 Å for both CITCO and MI-883 (Fig. 2e). It is also known that the free carboxylate of the C-terminus (S348) interacts with the K195’s side chain (on H4), which further stabilizes H12’s active conformation^6^. Interestingly, the short distance in both wtCAR/CITCO (median value: 3.5 Å) and wtCAR/MI-883 (3.8 Å) allows the formation of hydrogen bonds between K195 and S348, further stabilizing this region (Fig. 2f). Taken together, our results support a comparable affinity and potency of our novel compound MI-883 and CITCO in wtCAR.

- 1. **Conformational discrepancy of PXR-LBD-MI-883 interaction in comparison with classical agonist SR12813**

Trajectory analysis revealed a representative binding mode of SR12813 and MI-883 (Fig. 2g, h). The conformation of the PXR-LBD/MI-883 complex indicates that MI-883 is well accommodated in the LBP, where its 7-fluoroimidazo[1,2-*a*]pyridine moiety is oriented into the hydrophobic subpocket. Of note, this subpocket is crucial for different PXR ligands to interact, composed of a triad of hydrophobic amino acid residues; W299, F288, and Y306^7^. Our simulations suggest that both SR12813 and MI-883 share comparable π − π interactions with W299 and Y306 (~40%), whereas this interaction rises to ~70% with F288. In addition, MI-883 also has a hydrophobic contact with L209, which does not appear with SR12813.

The residue H407 has a critical role in ligand binding^8,9^, particularly agonist binding^10^. While H407 displays a stable H-bond with SR12813 (∼80%), only 24% of direct H-bond interaction occurs between MI-883 and H407 (Fig 2j). This difference could be related to the distinct H407 conformation in each of the two systems (Fig. 2g, h). Furthermore, MI-883 displays H-bond and water-mediated interactions to Q285 for ~50% and ~15%, respectively (Fig 2j). SR12813 lacks Q285 H-bond interaction but retains the S247 (~60% of the analyzed trajectory) water-mediated interaction. This interaction is observed in about 30% with MI-883. Meanwhile, R410 plays a role in stabilizing MI-883 as well. Overall, both ligands are stabilized by the highly similar hydrophobic interaction profile, whereas MI-883 shows additional polar interactions, except H407. To closely investigate the induced conformation resulting from the interaction profile, we pursued our study with locally assessing distance measurement. In NRs, αAF-2 (part of H3, H4, and H12) forms a suitable platform for the coregulators binding on the LBD surface^11^. Hence, we investigated the behavior of H12 in both systems, by monitoring the distance between H12 and H3 (Fig. 2k). This distance provides insight into the relative position of H12 to the LBD. In the presence of MI-883, this distance is slightly decreased (median of 10.3 Å) compared to what is observed for SR12813 (median values ∼11 Å) (Fig. 2k).

The H6 region is another region of interest for conformational investigation in PXR. It has been reported that the water channel between H2 and H6 could be an entry pathway for SR12813^12^. Our result shows that the distance between C207 (on H2′) and A312 (on β4-H6 loop) with MI-883 is smaller (12.1 Å) than that of PXR/SR12813 system (median 18.6 Å, Fig. 2l). This proposes the open conformation of β4-H6 in presence of SR12813, where this loop locates far from H2′ (Supplementary Fig. 8). By contrast, a closed conformation is preferred with MI-883, where the β4-H6 loop is closer to H2′.


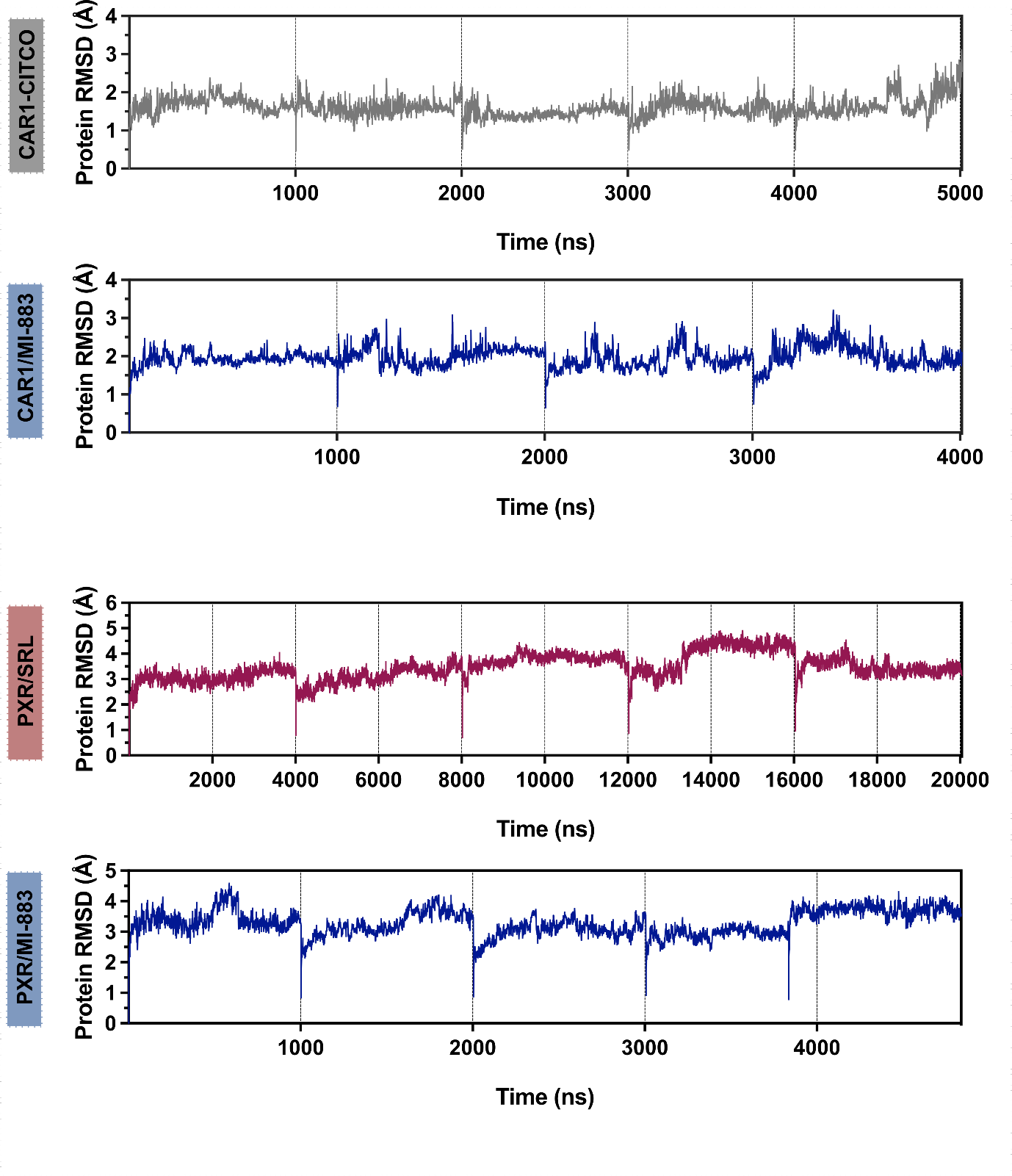


**Supplementary Figure 5**. Protein RMSD (Å) for each system over the course of the simulation. Herein, SRL refers to SR12813.


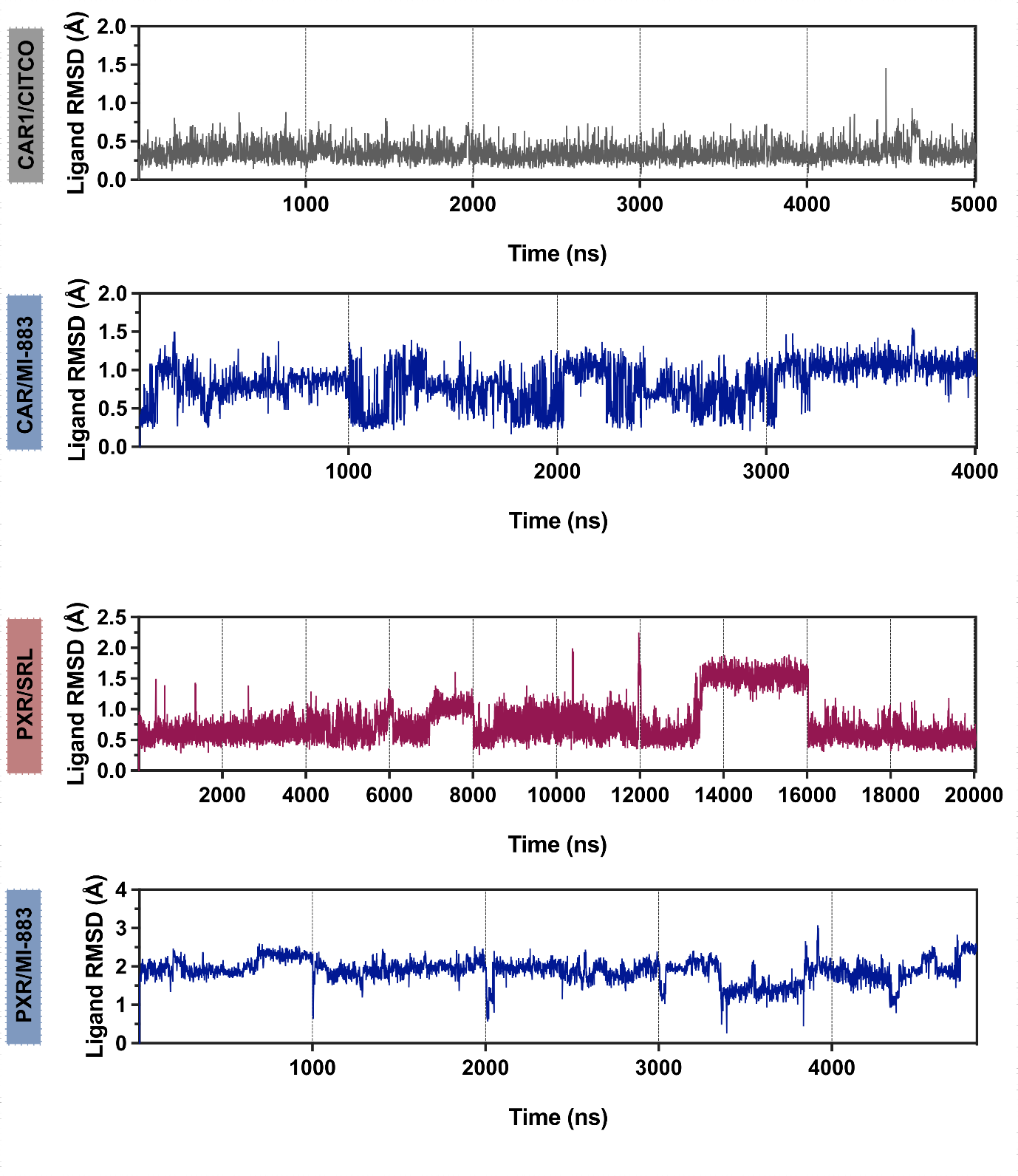


**Supplementary Figure 6**. Ligand RMSD (Å) for each ligand over the course of the simulation. Herein, SRL refers to SR12813.


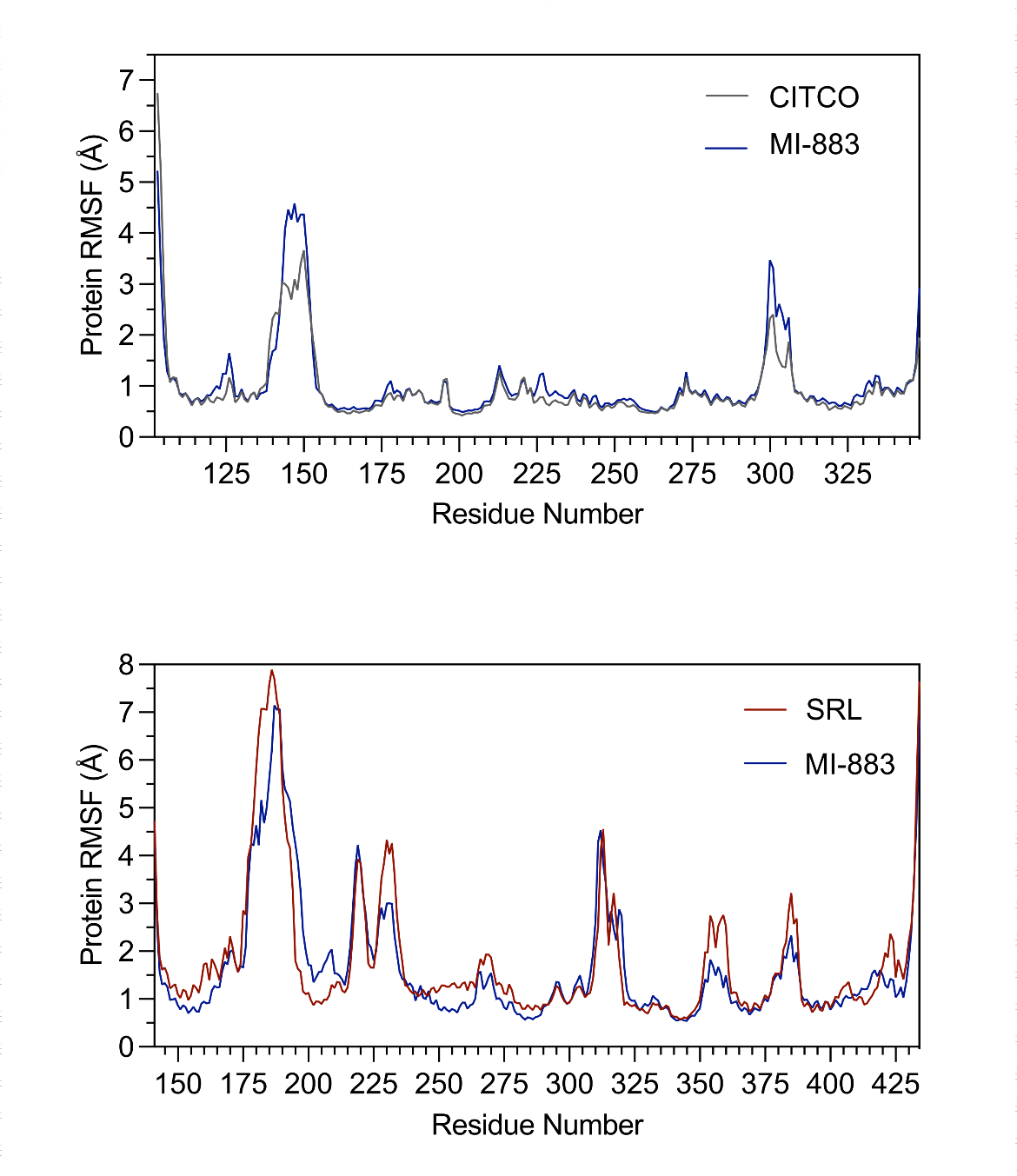


**Supplementary Figure 7**. Root-mean-square fluctuations (RMSF) of the proteins‘ backbone are shown with systems color-coded.


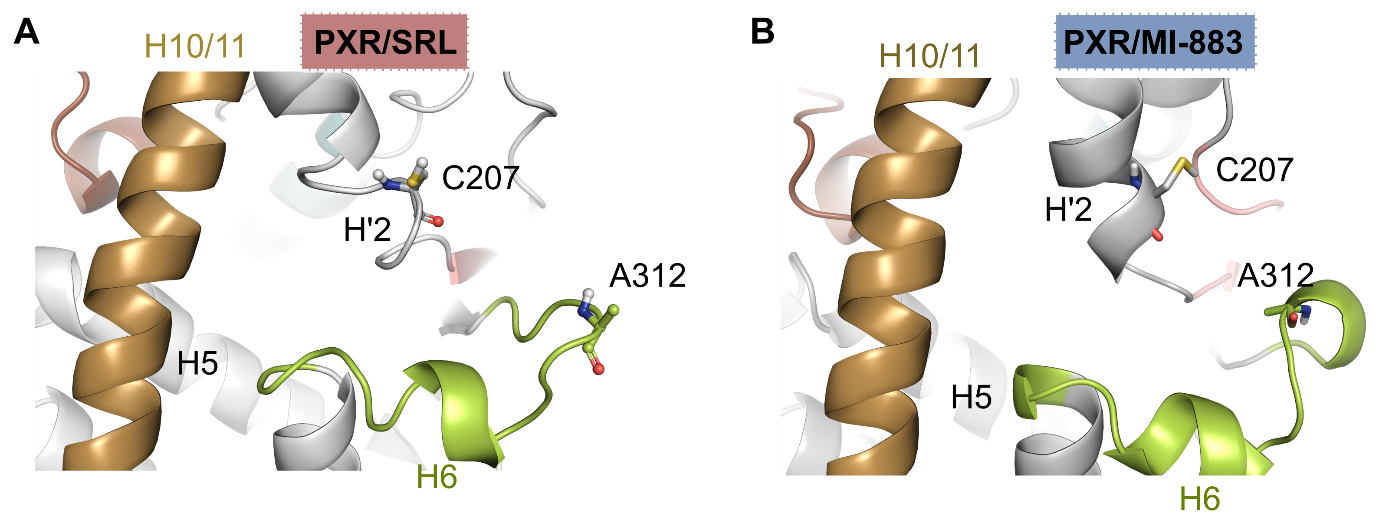


**Supplementary Figure 8**. The location of the H6 region within the PXR-LBD is depicted for SR12813 (SRL) (**a**) and MI-883 compound (**b**). The Cα-Cα distance between C207 (of H2’) and A312 (of β4-H6 loop) is depicted in stick representation. The configurations illustrate that the compound MI-883 promotes a closed configuration of β4-H6 loop. However, SRL, as a full PXR agonist SR12813, tends to result in more open conformations.

1. **Co-peptide recruitment (MARCoNI)**

The MI-883 and CITCO-modulated coregulator interactions with the HIS-tagged CAR-LBD (ThermoFisher, Cat. No PV4836) and anti-HIS antibody labeled with Alexa488 (Qiagen) was assessed using a PamChip microarray that contains 154 coregulator-derived binding peptides, including the LXXLL coactivator motif or the LXXXIXXXL corepressor motif from 66 diﬀerent coregulators (PamGene International B.V.)^13,14^.


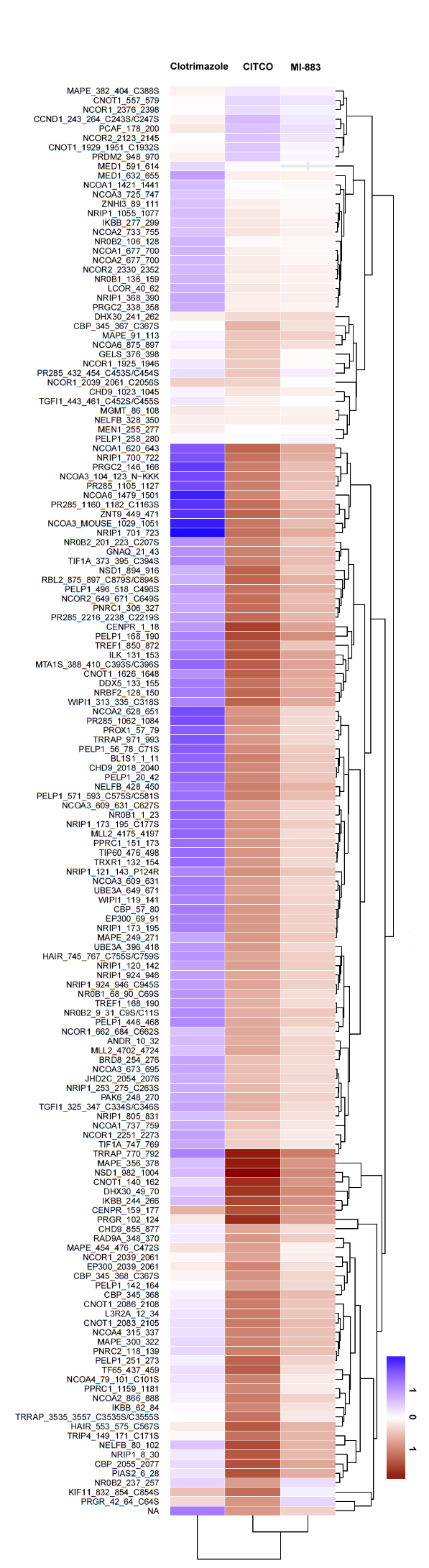


**Supplementary Figure 9.** MARCoNI dataset of CAR-coactivator/corepressor interactions in the presence of CITCO, MI-883, and clotrimazole (all at 100 μM). Modulation index (MI) is the log10-transformed relative binding value, which is calculated by the compound’s binding value, relative to the vehicle control (DMSO) binding value. Positive interaction indicates that ligands increase CAR–coregulator motif interactions, and the cut-oﬀ is MI>0; the binding value, interaction indicates that ligands decrease CAR-coregulator motifs interactions and the cut-oﬀ is MI<0; the binding value, >50; the relative binding value <1.


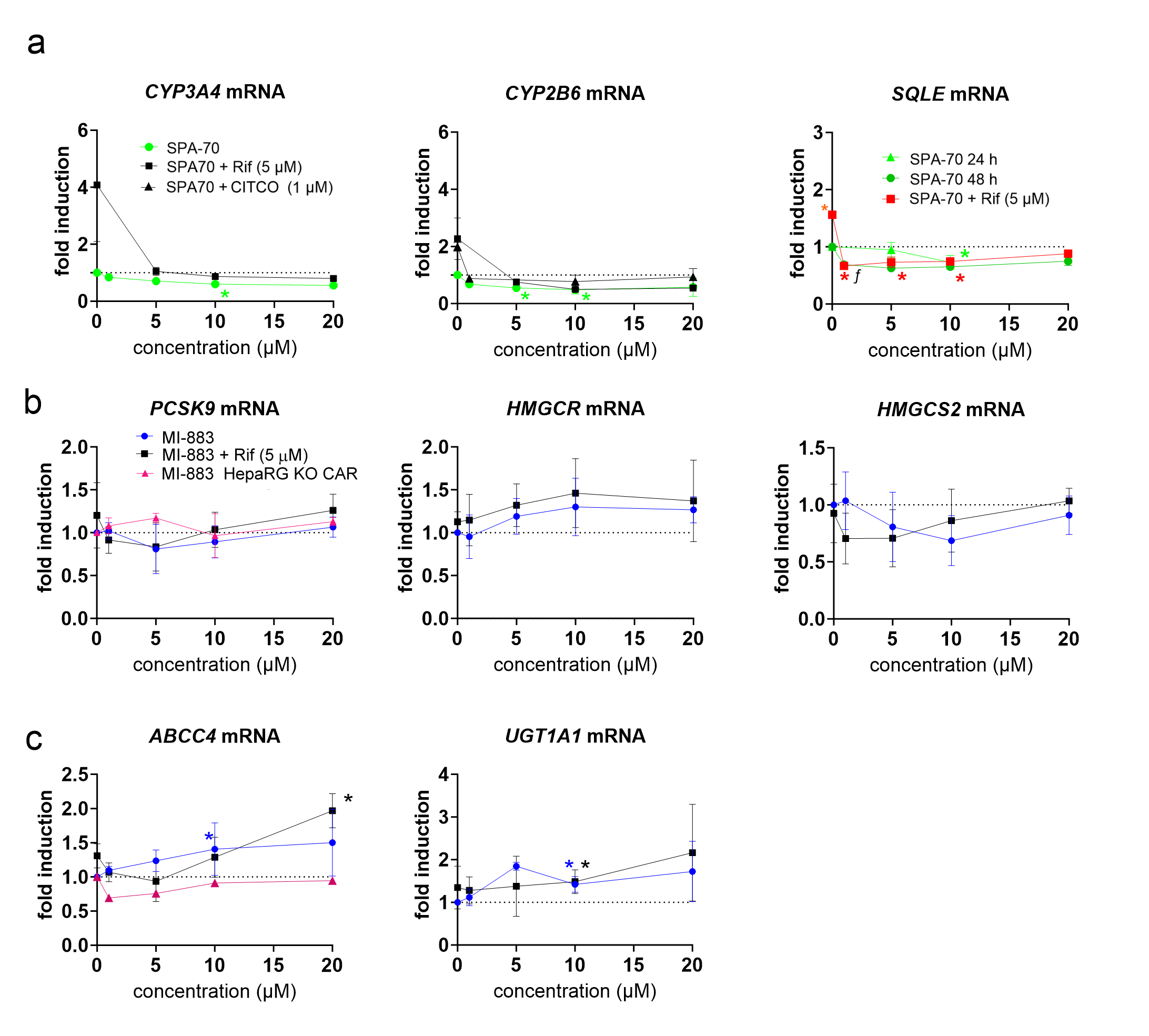


**Supplementary Figure 10.** Expression of *CYP3A4*, *CYP2B6*, *SQLE* (**a**), *PCSK9*, *HMGCR*, *HMGCS2* (**b**), *ABCC4,* and *UGT1A1* mRNA (**c**) in HepaRG and HepaRG KO CAR cells using RT-qPCR after SPA70 and MI-883 treatment. * *P* value <0.05 – statistically significant effect of SPA70 and MI-883 to control (vehicle-treated cells); ^ƒ^ *P* value<0.05 – statistically significant effect of SPA70 on rifampicin-mediated induction of SQLE mRNA (ANOVA with Dunnett’s test, n=3). Data represent means±S.D. from three independent experiments.

1. **Animal proof-of-concept experiments** **-plasma** **biochemistry measurement**

Blood was collected from the retro-orbital sinus of mice under isoflurane anesthesia into heparin-coated tubes (Kabe Laboratortechnik GmbH, Nümbrecht-Elsenroth, Germany) after 3 hours of fasting. Blood was left for 10 minutes at room temperature, and then kept on ice until centrifugation (5,000 g at 4°C for 10 minutes). The collected plasma was stored at -80 °C until measurement. Lipoprotein levels and other plasmatic parameters were measured on an AU480 biochemistry analyzer (Beckman Coulter, Brea, USA).

**
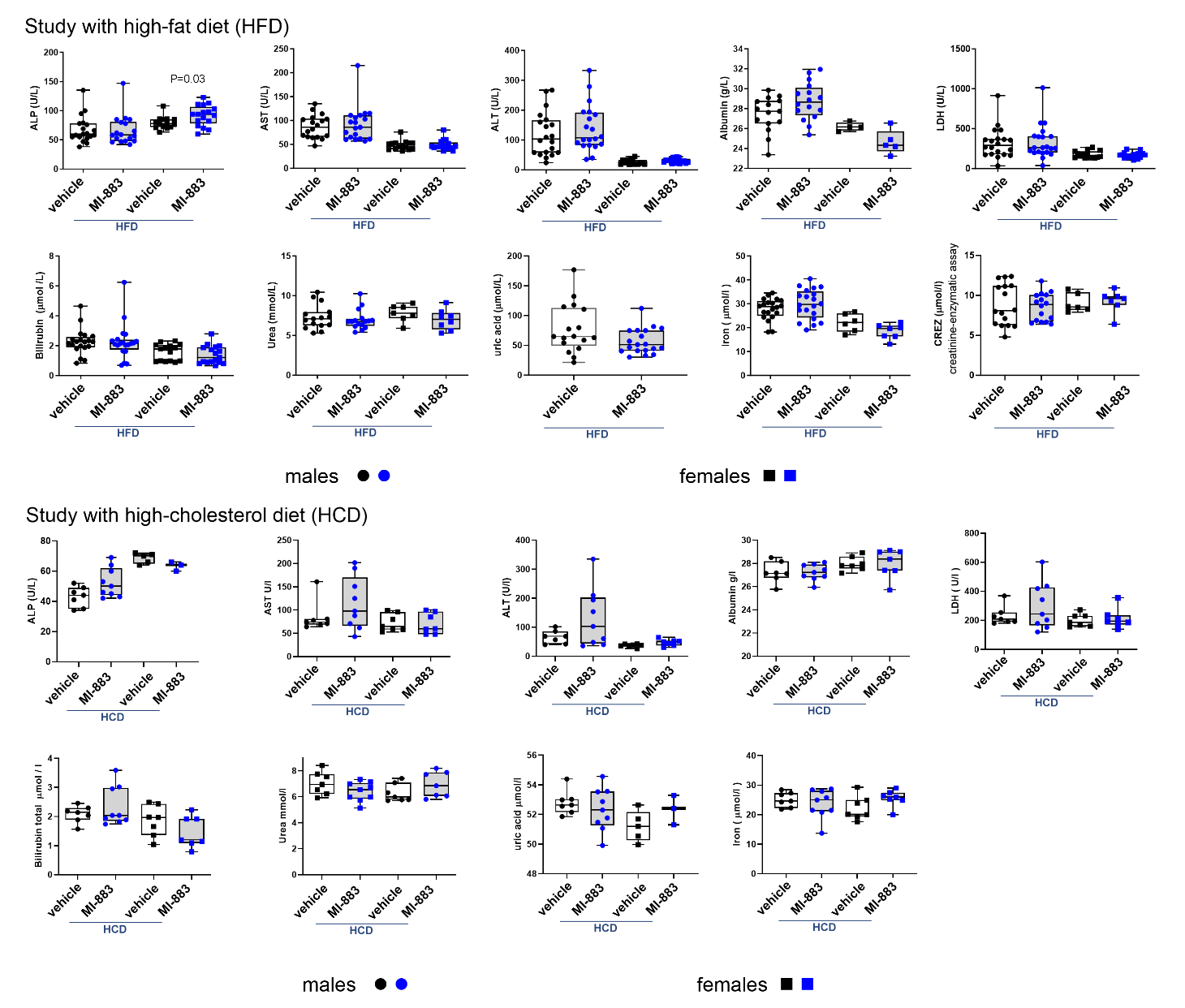
**

**Supplementary Figure 11.** Biochemical analyses of plasma in humanized PXR-CAR-CYP3A4/3A7 mice treated with MI-883 in proof-of-concept studies with high-fat diet (HFD)(n≥15) or high cholesterol diet (HCD)(n≥7). The box plots extend from the 25th to 75th percentiles. The line in the middle of the box is plotted at the median. Whiskers represent min to max values.

**
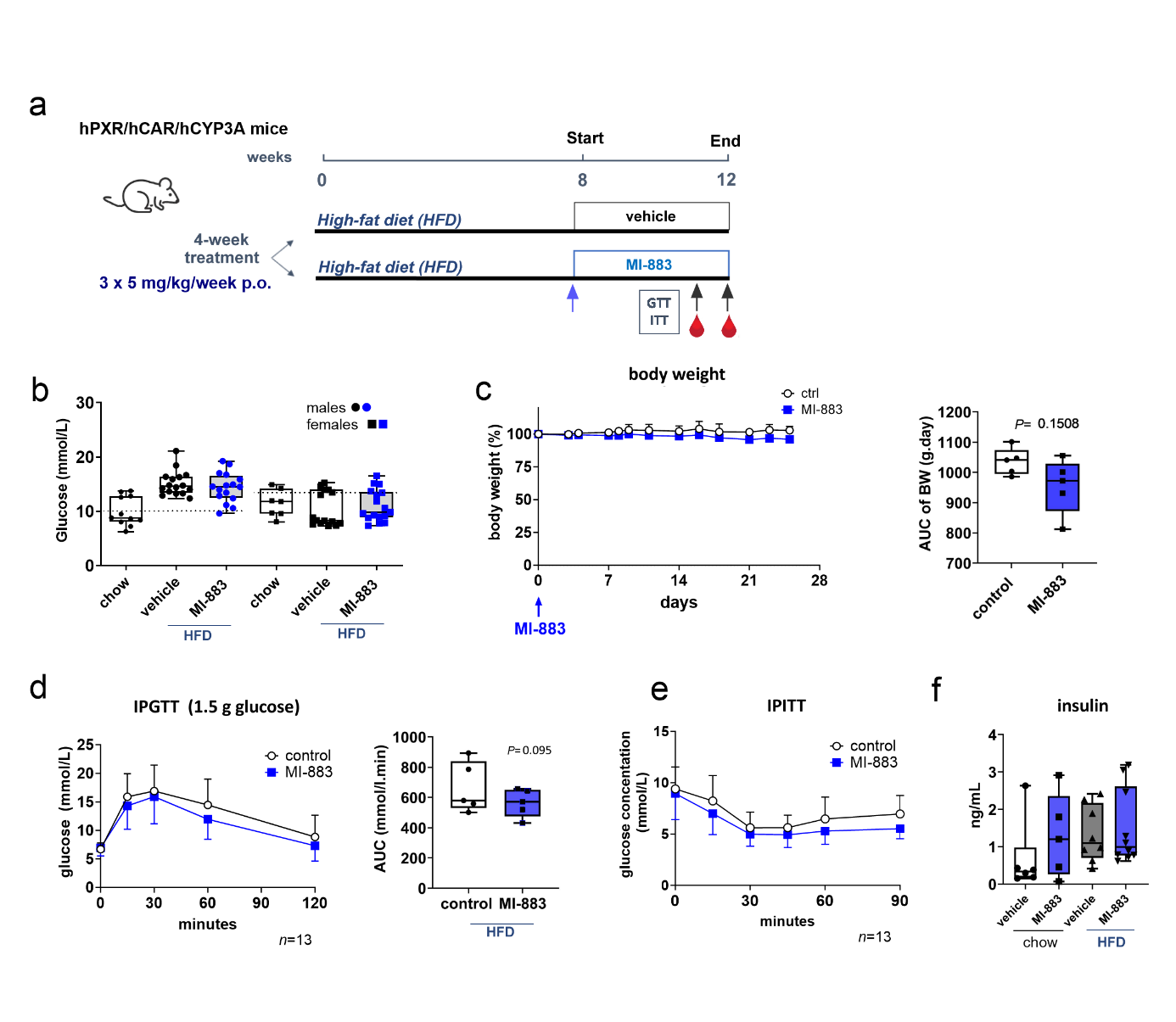
**

**Supplementary Figure 12.** (**a**) Effect of MI-883 on glucose metabolism in humanized PXR-CAR-CYP3A4/3A7 mice in the proof-of-concept study with high-fat diet (HFD). (**b**) Plasma glucose levels in both males and females. (**c**) Relative body weight gain (%) of male animals during the application of MI-883 in the study. (**d**) IPGTT and (e) IPITT tests were performed on male animals in the study. (**f)** plasma insulin concentration in male animals in the study with a high-fat diet (HFD) in comparison with animals on a chow diet. Box plots represent the median with 25% and 75% quartiles, whiskers represent minimum and maximum values. Data in panels **c**,**d,** and **e** are means±S.D.

**10.1.  Expression of genes involved in liver steatosis and triglyceride synthesis**

RT-qPCR expression studies were performed on liver samples from eleven humanized PXR-CAR-CYP3A4/3A7 mice that were fed a high-fat diet and treated with 5 mg/kg MI-883 three times per week for four weeks. Specific TaqMan probes were used, following the protocol described in Supplementary Table S3.


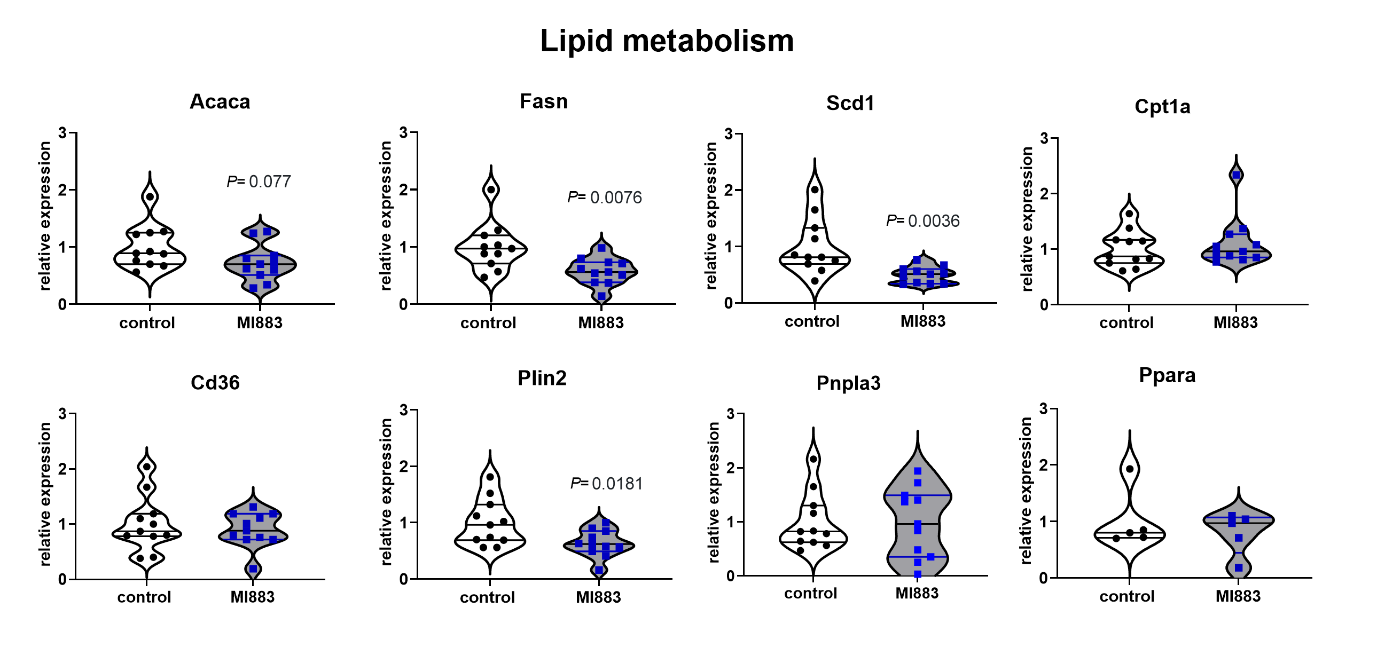


**Supplementary Figure 13.** Hepatic expression of genes involved in lipid and triglyceride metabolism in male PXR-CAR-CYP3A4/3A7 mice in the proof-of-concept study with high-fat diet (HFD) and treated with MI-883 (5 mg/kg 3x per week) (n=11). Expression in the MI-883 treated liver samples has been related to vehicle-treated liver samples (average set to 1). (Mann-Whitney U test). The violin plot represents the frequency distribution curve with the median and two quartile lines.

In the RT-qPCR reanalysis, MI-883 was found to affect the expression of three key enzymes involved in the synthesis of saturated and unsaturated fatty acids in the liver.

Fatty acid synthase (FASN) is a multifunctional enzyme that catalyzes the de novo synthesis of long-chain saturated fatty acids from acetyl-CoA and malonyl-CoA precursors. Acetyl-CoA carboxylase alpha (ACC/ACACA) catalyzes the carboxylation of acetyl-CoA to malonyl-CoA, which is the rate-limiting step in fatty acid synthesis. Stearoyl-CoA desaturase (SCD1) introduces the first cis double bond at the delta-9 position into saturated fatty acyl-CoA substrates, including palmitoyl-CoA and stearoyl-CoA. Additionally, perilipin 2, encoded by the PLIN2 gene, is located on the surface of intracellular lipid droplets, protecting them from lipolysis. Its activity is associated with hepatic lipid accumulation and steatosis^15^.

**10.2. Expression of other genes related to cholesterol absorption or FXR activation in the ileum**

RT-qPCR expression studies were conducted using humanized PXR-CAR-CYP3A4/3A7 mice that were fed a high-fat diet and treated with 5 mg/kg MI-883 three times per week for four weeks. Specific TaqMan probes were used to analyze ileal samples, following the protocol described in Supplementary Table S3*.*


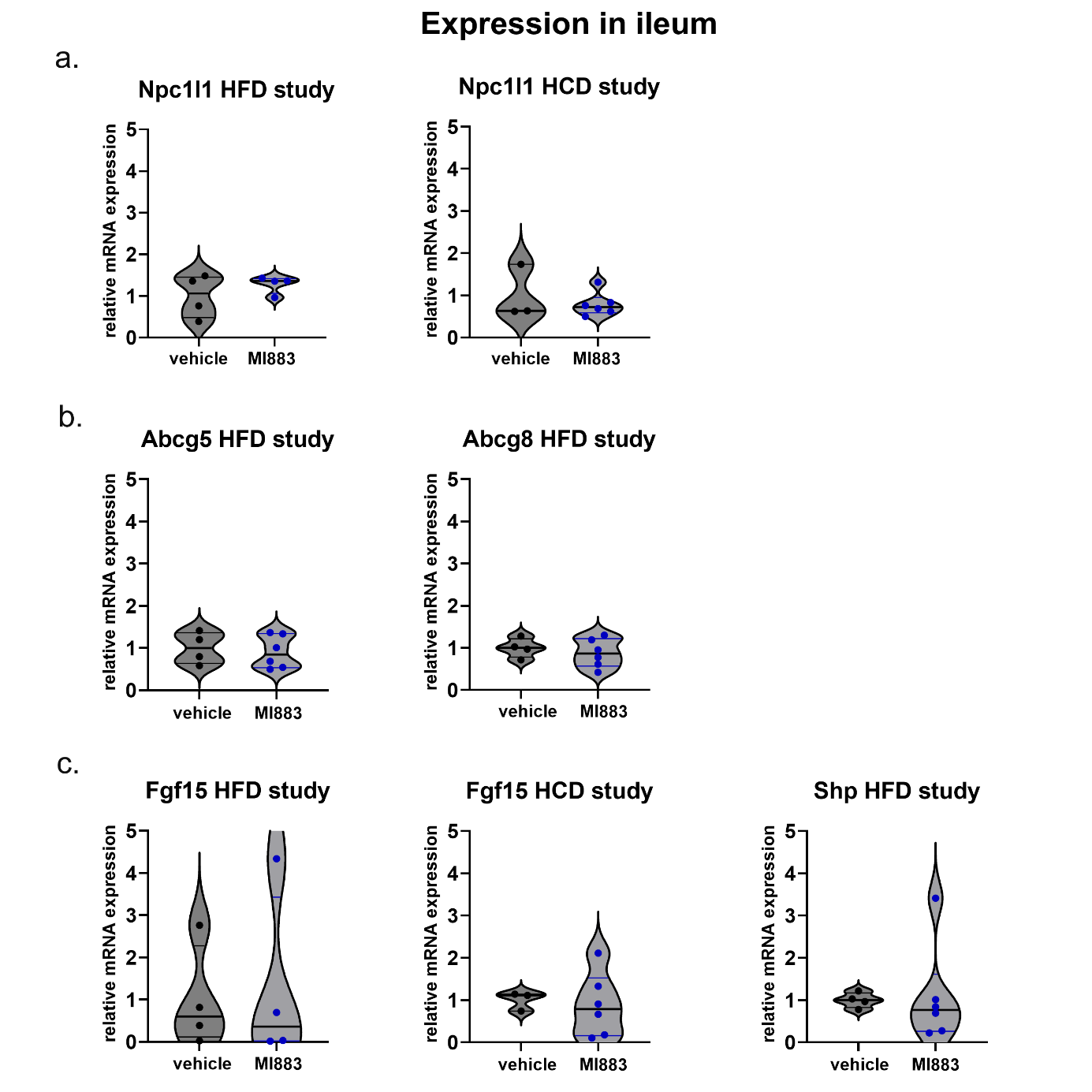


**Supplementary Figure 14.** mRNA expression of cholesterol transporters *Npc1l1* (**a**), *Abcg5*, and *Abcg8* (**b**) and *Fgf15* and *Nr0b2*(Shp) genes (**c**) in ileal samples from humanized PXR-CAR-CYP3A4/3A7 mice treated with MI-883 in studies with high-fat diet (HFD) or high cholesterol diet (HCD). RT-qPCR experiments have been performed with TaqMan probes and data are presented as relative fold up-/down-regulation to vehicle-treated animal samples (average set to be 1). The violin plot represents the frequency distribution curve with the median and two quartile lines.

We found that MI-883 has no effect on the mRNA expression of NPC1-like intracellular cholesterol transporter 1 (Npc1l1), which plays a key role in intestinal cholesterol absorption (Supplementary Fig. 14a). Additionally, we observed no regulation of *Abcg5*/*Abcg8* mRNA in the intestine after treatment with MI-883 (Supplementary Fig. 14b). *Abcg5* and *Abcg8* encode for heterodimer transporter that is an important cholesterol transporter involved in hepatobiliary and transintestinal cholesterol excretion (TICE) in the liver and intestine. Furthermore, the small heterodimer partner (Shp, *Nr0b2*), a sensitive target gene of FXR activation, and *Fgf15*, which encodes fibroblast growth factor 15—a negative regulator of bile acid biosynthesis—were also not significantly regulated (Supplementary Fig. 14c). Overall, these results indicate that MI-883 does not influence cholesterol absorption via Npc1l1 or the Abcg5/Abcg8 transporter in the ileum after treatment.

**11. The experiment with the PXB-mouse humanized liver mice treated with MI-883 – biochemistry analysis**

The clinical chemistry analyzer (BioMajestyTM Series JCA-BM6050, JEOL Ltd., Tokyo, Japan) was used to measure the blood h-Alb. LipoSEARCH to determine the serum level of total cholesterol, triglyceride, chylomicron, VLDL, LDL, and HDL was performed by SkyLight Biotech (Akita, Japan). Serum h-ALT1 concentration was determined based on an Enzyme-Linked ImmunoSorbent Assay (ELISA) developed by the Institute of Immunology Co., Ltd. (Tokyo, Japan). SpectraMax® ABS Plus Microplate Readers (Molecular Devices LLC, San Jose, CA, USA) were used for this measurement.

**
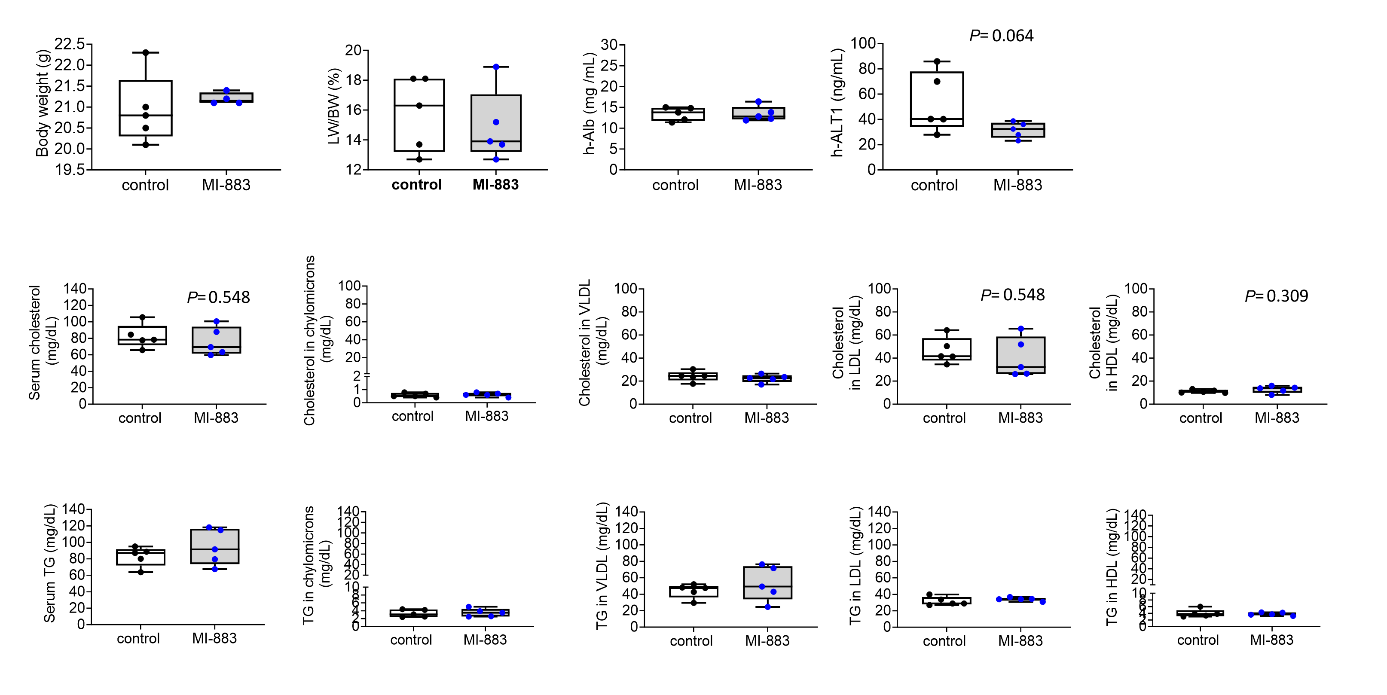
**

**Supplementary Figure 15.** Body weight, liver weight (LW), plasma human albumin (h-Alb), plasma human ALT (h-ALT1), cholesterol, and triglyceride levels in the study with PXB humanized liver mice treated with MI-883 (2.5 mg/kg every day for seven days)(n=5). Cholesterol and TG levels were evaluated in chylomicrons, VLDL, LDL, and HDL particles. Box plots represent the median with 25% and 75% quartiles, whiskers represent minimum and maximum values. (Mann-Whitney U test).

**12. Bile acid analysis in plasma, liver, and feces**

**12.1. Materials and reagents**

LC-MS grade methanol, acetonitrile, formic acid, acetic acid, and ammonium acetate (Honeywell, Riedel-de Haën, Germany) were purchased from AvantorScience (Prague, Czech Republic). Cholic acid (CA), ursodeoxycholic acid (UDCA), lithocholic acid (LCA), chenodeoxycholic acid (CDCA), deoxycholic acid (DCA), sodium glycochenodeoxycholate (GCDCA), sodium glycodeoxycholate (GDCA), sodium glycocholate (GCA), sodium tauroursodeoxycholate (TUDCA), sodium taurodeoxycholate (TDCA) and sodium taurocholate hydrate (TCA) were purchased from Merck (Prague, Czech Republic). Sodium glycoursodeoxycholate (GUDCA) and sodium taurochenodeoxycholate (TCDCA) were purchased from Steraloids (Newport, R.I., USA). Sodium glycolithocholate (GLCA) and sodium taurolithocholate (TLCA) were obtained from TRC Canada (Toronto, ON, Canada).

Deuterated internal standards of cholic acid-D5 (CA-D5), ursodeoxycholic acid-D4 (UDCA-D4), chenodeoxycholic acid-D4 (CDCA-D4), deoxycholic acid-D4 (DCA-D4), glycocholic acid-D4 (GCA-D4), glycochenodeoxycholic acid-D4 (GCDCA-D4), glycodeoxycholic acid-D4 (GDCA-D4), tauroursodeoxycholic acid-D4 (TUDCA-D4), glycoursodeoxycholic acid-D4 (GUDCA-D4), glycolithocholic acid-D4 (GLCA-D4), taurocholic acid-D4 (TCA-D4), taurochenodeoxycholic acid-D4 (TCDCA-D4), taurodeoxycholic acid-D4 (TDCA-D4) and taurolithocholic acid-D4 (TLCA-D4) were purchased from IsoScience (Ambler, PA, USA). MilliQ water was used throughout the study. Murideoxycholic acid (MDCA) was not analyzed.

**12.2. Sample preparation**

**12.2.1. Plasma**

To cover a wide concentration range of bile acids, two sets of plasma aliquots were prepared. The first set, with a volume of 10 μL, was diluted tenfold with MilliQ water. The second set, with a volume of 100 μL, was used without dilution, and the subsequent procedure remained identical for both sets. In each case, 100 μL of calibrator or plasma sample were taken, and 300 μL of HCOOH (1% v/v) in 100% acetonitrile containing internal standards (c = 167 nM) were added. The sample was vortex-mixed (IKA Vibrax for 60 s at 1500 RPM), allowed to stand for 10 minutes at -20°C, and then centrifuged at 13,000 × g (Eppendorf 5424R for 5 minutes at 20°C). A supernatant volume of 350 μL was transferred to a clean Eppendorf tube, and the solvent was evaporated to dryness under reduced pressure at a temperature of 60°C (Eppendorf Concentrator 5301). The dried samples were reconstituted in 100 μL of a solution containing 0.1% v/v HCOOH in 50% v/v acetonitrile, followed by vortex mixing (IKA Vibrax for 60 s at 1500 RPM) and centrifugation (Eppendorf 5424R at 13,000 × g for 1 minute at 20°C). The centrifuged samples were transferred to a 96-filter plate (AcroPrepTM Advance, wwPTFE) and filtered into a clean polypropylene 96-well plate (Waters, Milford, MA, USA).

**12.2.2. Liver, bile, and feces samples preparation**

Bile, liver homogenate, and stool samples have been sampled and prepared for analysis as we described recently^16^. Given the wide concentration range of bile acids in both bile and feces samples, we implemented sample dilution as follows: For bile samples, two aliquots of 10 μL each were prepared. The first aliquot was diluted tenfold by adding 90 μL of 50% (v/v) methanol, while the second aliquot underwent a 100-fold dilution by mixing it with 990 µL of 50% (v/v) methanol. A final volume of 100 μL was taken for subsequent processing. In the case of feces homogenate, a 50 μL aliquot was diluted twofold by adding 50 μL of 50% (v/v) methanol. This allowed us to prepare all samples with appropriate dilutions while maintaining a consistent volume of 100 μL. The subsequent processing steps were identical for both sample matrices.

To initiate the sample processing, 100 μL of the calibrator or diluted sample (bile or feces) was combined with 900 μL of a solution containing 1% formic acid (v/v) in 100% acetonitrile, supplemented with deuterated internal standards (c = 5 μM). The mixture underwent vortex mixing (using an IKA Vibrax for 60 seconds at 1500 RPM) and was allowed to stand for 10 minutes at -20°C. Following this, the sample was subjected to centrifugation at 13,000 × g (Eppendorf 5424R, 5 minutes, 20°C). After centrifugation, 100 μL of the supernatant was transferred to a clean Eppendorf tube. Subsequently, 900 μL of a solution containing 0.1% formic acid in 50% acetonitrile was added, and the resulting mixture was vortexed (IKA Vibrax, 60 sec, 1500 RPM). Following this step, 200 μL of the resultant solution was loaded into a 96-well filter plate (AcroPrepTM Advance, wwPTFE) and filtered into a clean polypropylene 96-well plate (Waters, Milford, MA, USA).

**
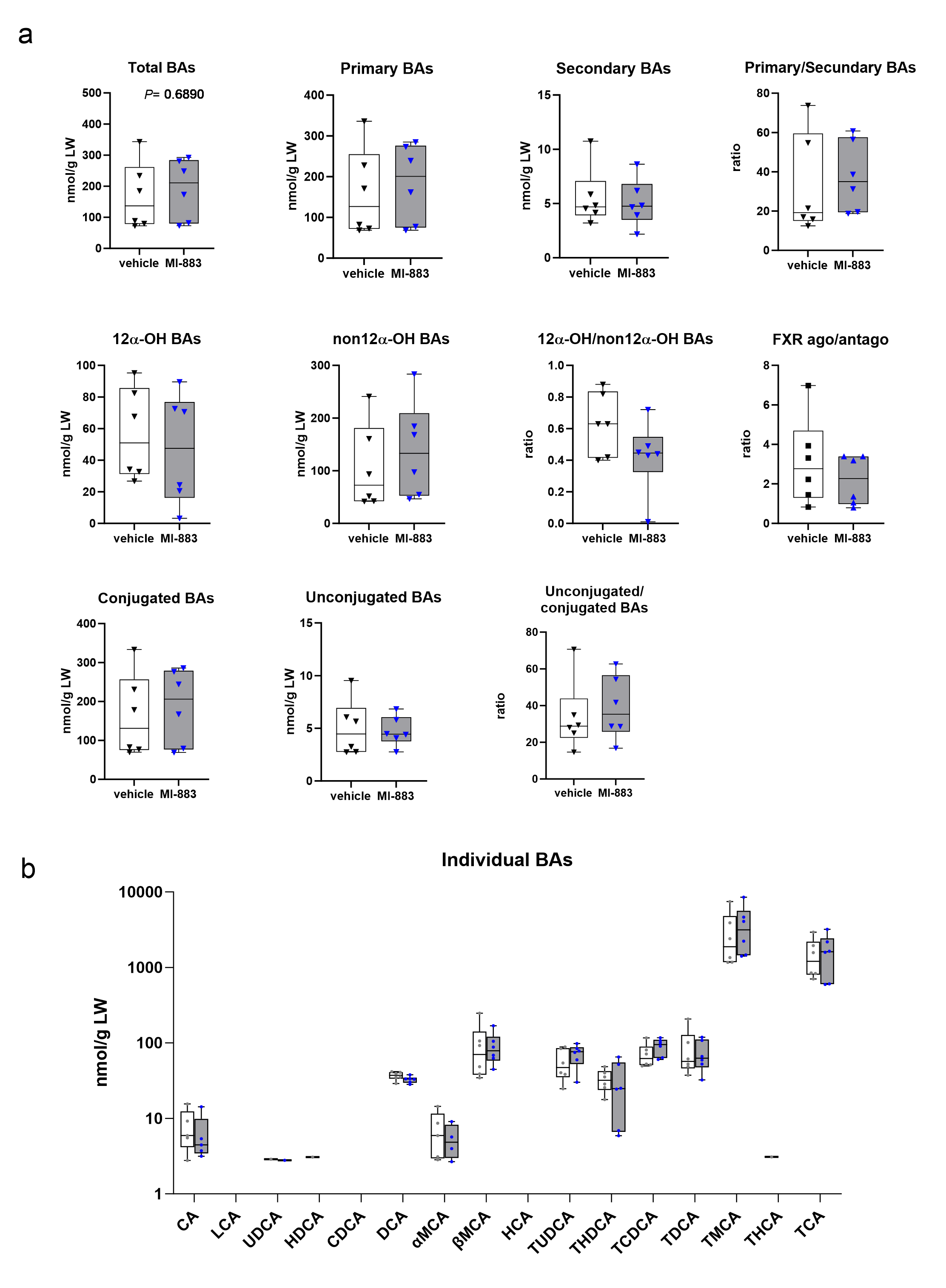
**

**Supplementary Figure 16.** Analysis of bile acid (BA) metabolome in the livers of humanized PXR-CAR-CYP3A4/3A7 male mice treated with MI-883 in the proof-of-concept study with HFD. (**a**) Comparison of total BAs content, primary BAs, secondary BAs, 12α-hydroxylated BAs, FXR activating BAs (chenodeoxycholic acid), unconjugated and conjugated BAs. (**b**) Individual BAs in the livers (n=6). Box plots represent the median (central line) with 25% and 75% quartiles, and whiskers represent minimum and maximum values.

**13. RT-qPCR** **and Western blotting**

RT-qPCR was used to examine gene expression in 2D and 3D PHH, HepaRG cells, or in mouse liver or intestine samples. Total RNA, reverse transcription, and qPCR were performed, and mRNA expression data was analyzed as we have described before^17,18^.

All qRT-qPCR experiments including Trizol reagent (Life Technologies) total RNA isolation, reverse-transcription using a random hexaprimer, and the MMLV Reverse Transcriptase Kit (ThermoFisher Scientific) we have described before^19^. qPCR was performed using TaqMan Fast Advanced Master Mix with TaqMan probes (ThermoFisher Scientific). Animal tissues (livers and ilea) have been snap-frozen in liquid nitrogen before RNA isolation. The delta-delta method was used for gene expression quantification normalized to at least two reference genes. Three technical replicates were used for each reaction considering the MIQE guidelines. Data are presented as fold induction to vehicle-treated cells/animals. TaqMan probes for murine and human genes are listed in Supplementary Table S3.

Western blotting experiments have been performed in BioRad instruments with BioRad precasted gels and reagents. Antibodies are listed in Supplementary Table S4. Densitometry has been done using ChemiDoc™ MP Imaging System and software (BioRad, Hercules, USA). Data are presented as relative protein expression to control samples. For protein expression quantification, areas in histograms have been calculated and related to a reference protein.

**Table S3.** TaqMan Gene Expression Assays (Life Technologies) for mouse and human genes used for RT-qPCR.

Catalog # 4331182  Assay ID Mm00839363_m1 Gene G6pc Mouse 116*

Catalog # 4331182 Assay ID Mm01247058_m1 Gene Pck1 Mouse 61

Catalog # 4331182 Assay ID Mm00662319_m1 Gene Fasn Mouse 67

Catalog # 4331182 Assay ID Mm00772290_m1 Gene Scd1 Mouse 60

Catalog # 4331182 Assay ID Mm00484150_m1 Gene Cyp7a1 Mouse 99

Catalog # 4331182 Assay ID Mm00501637_s1 Gene Cyp8b1 Mouse 69

Catalog # 4331182 Assay ID Mm00470430_m1 Gene Cyp27a1 Mouse 69

Catalog # 4331182 Assay ID Mm00484157_m1 Gene Cyp7b1 Mouse 62

Catalog # 4331182 Assay ID Mm00517066_m1 Gene CYp39a1 Mouse 95

Catalog # 4331182 Assay ID Mm00435123_m1 Gene Gadd45b Mouse 84

Catalog # 4331182 Assay ID Mm01278617_m1 Gene Mki67 Mouse 73

Catalog # 4331182 Assay ID Mm00448100_g1 Gene Pcna Mouse 117

Catalog # 4331182 Assay ID Mm00487804_m1 Gene Myc Mouse 89

Catalog # 4331182 Assay ID Mm01303209_m1 Gene Cdkn1a Mouse 62

Catalog # 4331182 Assay ID Mm01257352_g1 Gene Mcl-1 Mouse 64

Catalog # 4331182 Assay ID Mm00487656_m1 Gene Mdm2 Mouse 113

Catalog # 4331182 Assay ID Mm00432050_m1 Gene Bax Mouse 68

Catalog # 4331182 Assay ID Mm00477631_m1 Gene Bcl2 Mouse 85

Catalog # 4331182 Assay ID Mm00443947_m1 Gene Cdk2 Mouse 62

Catalog # 4331182 Assay ID Mm01303209_m1 Gene Cdkn1a Mouse 62

Catalog # 4331182 Assay ID Mm00522599_m1 Gene Meg3 Mouse 65

Catalog # 4331182 Assay ID Mm00456591_m1 Gene Cyp2b10 Mouse 132

Catalog # 4331182 Assay ID Mm00456588 Gene Cyp2b10 Mouse 88

Catalog # 4331182 Assay ID Mm00488258_m1 Gene Slc10a2 Mouse 76

Catalog # 4331182 Assay ID Mm00521530_m1 Gene Slc51a Mouse 65

Catalog # 4331182 Assay ID Mm01175040_m1 Gene Slc51b Mouse 90

Catalog # 4331182 Assay ID Mm00441421_m1 Gene Slc10a1 Mouse 85

Catalog # 4331182 Assay ID Mm00440761_m1 Gene Abcb1a Mouse 82

Catalog # 4331182 Assay ID Mm00496899_m1 Gene Abcc2 Mouse 64

Catalog # 4331182 Assay ID Mm00551550_m1 Gene Abcc3 Mouse 79

Catalog # 4331182 Assay ID Mm01226372_m1 Gene Abcc4 Mouse 60

Catalog # 4331182 Assay ID Mm00445168_m1 Gene Abcb11 Mouse 100

Catalog # 4331182 Assay ID Mm00655373_m1 Gene Ugt2b34 Mouse 113

Catalog # 4331182 Assay ID Mm07306663_mH Gene Ugt1a1 Mouse 70

Catalog # 4331182 Assay ID Mm04205659_mH Gene Sult2a1 Mouse 110

Catalog # 4331182 Assay ID Mm00550338_m1 Gene Srebf1 Mouse 62

Catalog # 4331182 Assay ID Mm01304569_m1 Gene Hmgcs1 Mouse 87

Catalog # 4331182 Assay ID Mm00463389_m1 Gene Insig1 Mouse 117

Catalog # 4331182 Assay ID Mm99999915_g1 Gene Gapdh Mouse 109

Catalog # 4331182 Assay ID Mm03024075_m1 Gene Hprt Mouse 131

Catalog # 4331182 Assay ID Mm00437762_m1 Gene B2m Mouse 77

*Amplicon

Table S3. To be continued -human genes

Catalog # 4331182 Assay ID Hs00604506_m1 Gene CYP3A4 Human 119

Catalog # 4331182 Assay ID Hs04183483_g1 Gene CYP2B6 Human 63

Catalog # 4331182 Assay ID Hs00978452_m1 Gene ABCC3 Human 64

Catalog # 4331182 Assay ID Hs00988721_m1 Gene ABCC4 Human 141

Catalog # 4331182 Assay ID Hs02511055_s1 Gene UGT1A1 Human 134

Catalog # 4331182 Assay ID Hs00234219_m1 Gene SULT2A1 Human 98

Catalog # 4331182 Assay ID Hs00540450_s1 Gene MDM2 Human 104

Catalog # 4331182 Assay ID Hs00427214_g1 Gene PCNA Human 138

Catalog # 4331182 Assay ID Hs01032443_m1 Gene MKI67 Human 66

Catalog # 4331182 Assay ID Hs00169587_m1 Gene GADD45B Human 74

Catalog # 4331182 Assay ID Hs01073586_m1 Gene FOXM1 Human 77

Catalog # 4331182 Assay ID Hs00167982_m1 Gene CYP7A1 Human 66

Catalog # 4331182 Assay ID Hs01088679_g1 Gene SREBF1 Human 84

Catalog # 4331182 Assay ID Hs01088691_m1 Gene SREBF1 Human 90

Catalog # 4331182 Assay ID Hs01081784_m1 Gene SREBF2 Human 91

Catalog # 4331182 Assay ID Hs01123768_m1 Gene SQLE Human 109

Catalog # 4331182 Assay ID Hs00356479_g1 Gene INSIG1 Human 93

Catalog # 4331182 Assay ID Hs00545399_m1 Gene PCSK9 Human 109

Catalog # 4331182 Assay ID Hs01092524_m1 Gene LDLR Human 75

Catalog # 4331182 Assay ID Hs00168352_m1 Gene HMGCR Human 67

Catalog # 4331182 Assay ID Hs00985427_m1 Gene HMGCS2 Human 91

Catalog # 4331182 Assay ID Hs00609178_m1 Gene G6PC Human 123

Catalog # 4331182 Assay ID Hs00159918_m1 Gene PCK1 Human 81

Catalog # 4331182 Assay ID Hs02800695_m1 Gene HPRT1 Human 82

Catalog # 4331182 Assay ID Hs02758991_g1 Gene GAPDH Human 93

Catalog # 4331182 Assay ID Hs00427620_m1 Gene TBP Human 91

**Table S4.** Primary and secondary antibodies used for Western blot analysis.

| **Protein** | **Source** | **dilution** | **secondary antibody dilution** |
| --- | --- | --- | --- |
| CYP7A1 | MABD42; Anti-Cyp7a1 Antibody, clone 15B9.1 (Sigma-Aldrich/Merck) | x1:2,000 | x1:5,000 (NA931, Amersham ECL Mouse IgG, HRP-linked whole Ab (from sheep), Cytiva) |
| CYP8B1 | PA5-37088; CYP8B1 Polyclonal Antibody 100 µl, (Thermo Fisher Scientific) | x1:1,000 | x1:200,000 (Goat anti-rabbit IgG (H+L) secondary antibody, HRP Invitrogen Thermo Fisher Cat. No#31460, used for other antibodies) |
| HMGCR | HMGCR Polyclonal Antibody Catalog # PA5-37367 (Invitrogen, Thermo Fisher Scientific) | x1: 1,000 | x1: 200,000 |
| MRP4 | PA5-78695; MRP4 Polyclonal Antibody 100 µg, (Thermo Fisher Scientific) | x1: 2,000 | x1: 200,000 |
| Slc10a2 | PA5-116296; SLC10A2 Polyclonal Antibody) (Thermo Fisher Scientific) | x1:1,000 | x1:2,000 |
| SREBP1 | PA1-337; SREBP1 Antibody (Thermo Fisher Scientific)  Synthetic peptide corresponding to residues M(32) L Q L I N N Q D S D F P G L F(47) of mouse SREBP 1 | x1: 1,000 | x1: 200,000 |
| Cyp2b6 | AB9916, Anti-CYP2b10 Antibody serum, Chemicon® Sigma-Aldrich (Merck) | x1:5,000 | x1:200,000 |
| CYP3A4 | PA1-343; CYP3A4 Polyclonal Antibody (Thermo Fisher Scientific) | x1:2,000 | x1:200,000 |
| SREBP2 | PA5-88943; Invitrogen SREBP2 Polyclonal Antibody (ThermoFisher Scientific) | x1:1,000 | x1:200,000 |
| INSIG1 | PA5-100150; INSIG1 Polyclonal Antibody (Thermo Fisher Scientific) | 1:1,000 | x1:200,000 |
| Beta-actin | MA5-32540; beta Actin Recombinant Monoclonal Antibody (JF53-10), HRP (Thermo Fisher Scientific) | x1:2,000 | none |

**
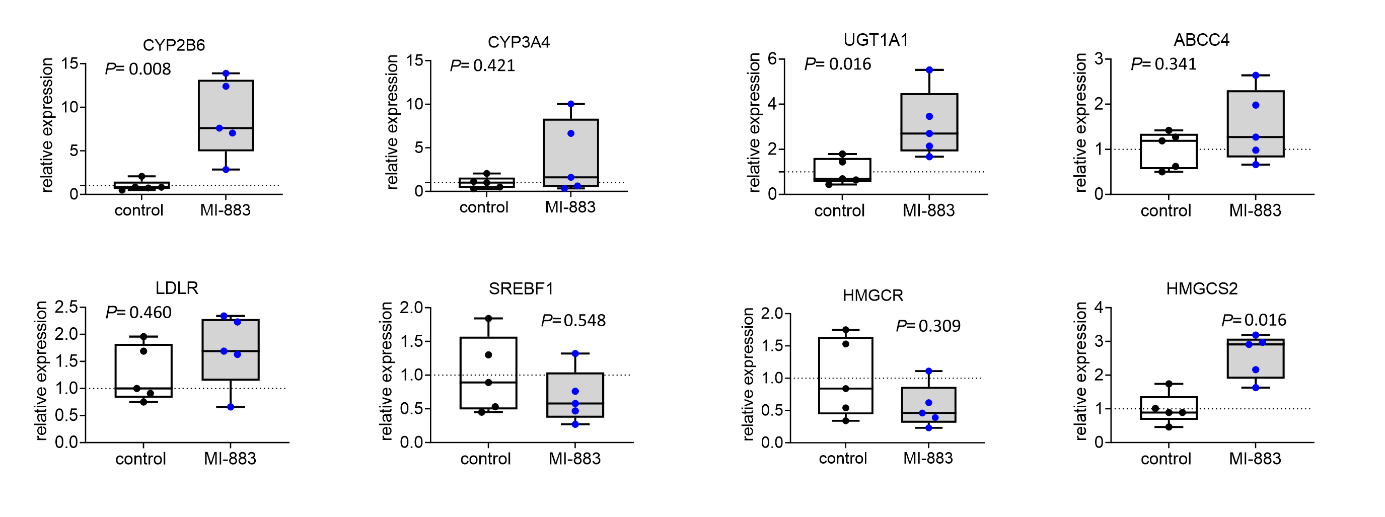
**

**Supplementary Figure 17.** Expression of selected genes in PXB humanized liver mice after one week of treatment with MI-883 (2.5 mg/kg every day for seven days) in the livers. RT-qPCR analysis has been performed with TaqMan probes as indicated in Table S3. Box plots represent the median (line) with 25% and 75% quartiles, and whiskers represent minimum and maximum values. (Mann-Whitney U test).

**14. Cholesterol, C4, and lathosterol determination**

**14.1. Preparation of liver homogenates**

Liver tissue (25-80 mg fresh weight) was diluted 10x with PBS (by weight), cut into small pieces using scissors, and treated with multiple (10-20) short pulses of ultrasound until a smooth homogenate was obtained. This 10% liver sonicate was used in subsequent analyses.

**14.2. Cholesterol determination**

Cholesterol (total and free) was measured using GC-MS according to Cohen *et al* ^20^. Twenty µL of 10% liver sonicate, approximately 2 µg of internal standard (d7-cholesterol, Sigma Aldrich), and 6 mL of ethanol (p.a. Penta) were thoroughly mixed and split into two equal parts. The first served for the determination of total cholesterol, while the other was used to measure free cholesterol. Cholesteryl esters were hydrolyzed (by adding 390 µL of KOH (Penta), 8.9 M and incubating for at least 4 hours at 37°C) in the first part, while this step was omitted in the other. Next, 2 mL of water and 5 mL of hexane (LiChrosolv, Merck) were added, vortex mixed and centrifuged for 5 minutes (2500 g, room temperature). The hexane phase was evaporated and treated with 50 µL of derivatization mixture (pyridine:N,O-bis(trimethylsilyl)acetamide:chlorotrimethylsilane=6:2:1, all from Sigma) for 30 minutes at 37°C. One µL of the mixture was injected (split 1:10) into the GC-MS system (Agilent 8890 GC System and Agilent 7000D GC/TQ), equipped with J&W HP-5ms Ultra Inert column (30 m x0.25 mm, 0.25 µm, Agilent) with helium (2mL/min) as a mobile phase. The temperatures were as follows: injection port 280°C; column 200 °C (0- 1 min), 200-300°C 1-5 min, 300°C (5-13 min). Cholesterol concentration was calculated based on the ratios of cholesterol/d-7 cholesterol derivatives (at *m/z* 458.4 and 465.4, respectively).

Lathosterol was measured using the same procedure as for free cholesterol with slight modifications: the volume of liver homogenate as well as the injection volume were increased (100 µL and 2 µL, respectively) while the amount of internal standard was reduced to approximately 100 ng. Also, the mixture was sonicated for 10 minutes after adding ethanol.

**14.3. C4 determination**

Liver sonicate (10%, 200-500 mg) was mixed with 1 ng of internal standard (d7-C4, Santa Cruz Biotechnology), brought to 1 mL with saline and solid phase extracted as described previously ^21^. Final eluate was evaporated, dissolved in 80 µL 75% methanol (LC-MS, Biosolve), and 20 µL were analyzed using LC-MS/MS as described previously^22^.

**14.4. Protein quantification**

Liver sonicate (10%) was further diluted 30x (by weight) and 5 µL was analyzed using DC Protein Assay (Bio-Rad) according to manufacturer instructions (microassay format). Bovine serum albumin was used as standard. All samples were analyzed in triplicates.

**15. Liver Enzymatic TG measurement**

The liver tissue (10 mg) was homogenized using a pre-cooled steel bead in 225 μL of methanol in TissueLyser II (30 Hz, 2 × 30 s) (Qiagen, Hilden, Germany). This was followed by the addition of 750 μL of ice-cold methyl tert-butyl ether. Samples were vortexed and incubated for 1 h on a rotating wheel. Next, 188 μL of LC/MS-grade water was added and, after vortexing, the mixture underwent a short incubation and centrifugation (14000 × g, 4 °C, 3 min). The upper organic phase containing lipids was collected and 300 μL was transferred to a vial containing 40 μL of 10% Triton X-100 in methanol, followed by evaporation on a heating block with shaking function (300 rpm, 45 °C). LC/MS-grade water (100 μL) was added to the evaporated sample and the tube was vortexed (1000 rpm, 35 °C). The samples were measured using a TG assay (Beckman Coulter, Brea, CA, USA) and a glycerol assay (Randox laboratories, Crumlin, UK) on an AU480 biochemistry analyzer (Beckman Coulter, Brea, CA, USA). The glycerol value was subtracted from the TG value. Livers from seven (n=7) vehicle-treated controls and ten (n=10) MI-883-treated animals were used.

**16.** **Mass Spectrometry analysis of lipids - Lipidomics**

**16.1. Material – solvents, additives, and internal standards**

LC-MS grade solutions and additives, such as acetonitrile, methanol, 2-propanol, hexane, water, ammonium carbonate, and ammonium acetate (Honeywell, Riedel-de Haën, Germany) were purchased from Thermo Fisher Scientific (Waltham, MA, USA). Chloroform (Lichrosolv) was purchased from Merck (Darmstadt, Germany), and supercritical carbon dioxide (scCO_2_) with 99.995% purity from Messer (Bad Soden, Germany). Deionized water) was obtained from the Milli-Q water purification system (Millipore, Molsheim, France). Lipid class internal standards, such as 15:0-18:1-D7 phosphatidylcholine (**PC 33:1 D7**), 18:1-D7 lysophosphatidylcholine (**LPC 18:1 D7**), 15:0-18:1-D7 phosphatidylethanolamine (**PE 33:1 D7**), 14:0 lysophosphatidylethanolamine (**LPE 14:0**), 18:1-D9 sphingomyelin (**SM 36:2 D9**), 15:0-18:1-D7-15:0 triacylglycerol (**TG 48:1 D7**), 15:0-18:1-D7 diacylglycerol (**DG 33:1 D7**), 18:1-D7 monoacylglycerol (**MG 18:1 D7**), 16:0 cholesteryl-D7 ester (**CE 16:0 D7**), d18:1-D7/18:0 ceramide (**Cer 36:1;O2 D7**), manufactured by Avanti Polar Lipids (Alabaster, AL, USA) were purchased from Merck.

**16.2. Sample preparation**

25 mg of liver tissue was homogenized using mortar and pestle. This was followed by addition of 20 µL of internal standard mixture and 6ml of chloroform/methanol (2:1, v/v). After 10 seconds of vortexing and ultrasonication (15 min, 40°C), 1.2ml of aqueous ammonium carbonate (250 mM) was added. After 10s of vortexing, ultrasonication (15min, 40°C) and centrifugation (3,000 rpm, 3min), organic phase was transferred to new vial and 2ml of chloroform were added to the aqueous phase. After 10s of vortexing, ultrasonication (15min, 40°C) and centrifugation (3,000 rpm, 3 min), organic phase was removed and combined with the previous one. Collected organic phase was evaporated under nitrogen flow and then dissolved in 500 µL of chloroform/methanol (1:1) mixture. After further dilution (5x) by chloroform/methanol (1:1), the sample was injected into UHPSFC/MS system for analysis.

**16.3. UHPSFC/MS measurement**

Lipid separation using UHPSFC (Acquity UPC^2^ instrument from Waters; Milford, MA, USA) was performed on column Viridis BEH (100 × 3 mm, 1.7 µm) with the following conditions: 60 °C column temperature, 1.9 mL/min flow rate, and 1 µL injection volume. The injection needle was washed with hexane/2-propanol/water (2:2:1, *v/v/v*) after each injection. The following linear gradient was performed using scCO_2_ and methanol (30 mM ammonium acetate + 1% of water) used as a modifier: 0 min – 1% modifier, 1.5 min – 16% modifier, 4 min – 51% modifier, 7 min – 51% modifier, 7.51 min – 1% modifier, and the equilibration with the total run time of 8 min. The automatic back-pressure regulator (ABPR) was set to 1,800 psi and the autosampler temperature to 4°C. Methanol with 30 mM ammonium acetate and 1% of water was used as the make-up solvent with a flow rate of 0.25 mL/min. The SFC was connected with the hybrid quadrupole - time of flight (QTOF) mass spectrometer Synapt G2-Si from Waters with the following conditions: sensitivity mode applying positive ESI mode, the mass range of *m/z* 150-1200, the capillary voltage of 3 kV, the sampling cone of 20 V, the source offset of 90 V, the source temperature of 150 °C, the desolvation temperature of 500 °C, the cone gas flow of 50 L/h, the desolvation gas flow of 1000 L/h, and the nebulizer gas flow of 4 bar. Mass spectra were acquired in the continuum mode, with a scan time of 0.5 seconds and the peptide leucine enkephalin as the lock mass.

**17. Genotoxicity testing** - **reverse-mutation Salmonella typhimurium Ames test**

The mutagenic activity of MI-883 was detected using the commercially available Muta-ChromoPlate bacterial strain kit (ebpi, Mississauga, Ontario, Canada), which is a 96-well microplate version of the reverse-mutation *Salmonella typhimurium* Ames test with S9 rat-liver extract. *Salmonella typhimurium tester strains TA 98* (detection of frameshift mutagens) and *TA 100* (detection of base-exchange mutations) were used as we described before^1^.

Consistently, Bacterial Reverse Mutation Test was performed as a service at Research Institute for Organic Syntheses Inc., (Pardubice, Czech Republic) in *Salmonella typhimurium* strains TA 98, TA 100, TA 1535, TA 1537, and one indicator *Escherichia coli WP2 uvrA* strains. The report is available after request.

Conclusion: “Under the above-described experimental design, the test item, MI-883, was nonmutagenic for all the used indicator strains in experiments with and without metabolic activation.”

**18. NOAEL-Repeated dose 28-day oral toxicity study in rats (Non – GLP Study)**

A repeated dose 28-day oral toxicity study in Rats (Non–GLP Study) was carried out according to OECD Guideline for Testing of Chemicals No. 407 and relevant Test Facility SOPs at the Institute of Physiology, Academy of Sciences, Czech Republic as a service. The report is available after request. Conclusion: “No clinical signs of toxicity were observed in the animals of all dose groups treated repeatedly *per os* by MI833 for consecutive 28 days. No clinical changes were observed during the administration period or the recovery period in the two selected groups. MI833, orally administered to rats in the highest dose of 10 mg/kg using two groups did not cause gross or histopathological changes compared to the control group. NOAEL is defined as the highest dose level that does not produce a significant increase in adverse effects in comparison to the control group. Based on the results of this study, it can only be stated that the NOAEL is higher than 10 mg/kg under conditions used in this study.”

**19. Metabolomic analysis of plasma from mice treated with MI-883 and fed high-fat diet (HFD)**

Metabolomic plasma analysis was performed using plasma samples from male PXR/CAR/CYP3A4 mice (n=5) treated with MI-883 in the proof-of-concept study involving a high-fat diet^23^. Plasma samples were prepared on ice, and each sample was separated into two 20 μl aliquots. All prepared aliquots were then frozen and stored at −80°C until analysis. Plasma aliquots were thawed, and protein precipitation was performed by adding 60 μl of a mixture containing 40% methanol, 40% acetonitrile, and 20% water. Samples were mixed, incubated for 12 hours at -20°C, and then centrifuged at 20,000 g for 10 minutes. The supernatants were collected for analysis by LC-MS/MS. A pooled quality control sample was prepared by mixing 10 μl from each sample. LC separation was performed on an iHILIC® Classic column (iHILIC®-(P) Classic, HILIC Column, PEEK,100x2.1mm, 5µm, 200Å, Hilicon, Umeå, Sweden) using a water/acetonitrile gradient with 10 mM ammonium formate, starting from 95% to 42% over 15 minutes in both polarities. LC-MS/MS analysis was conducted on the ID-X Orbitrap Tribrid Mass Spectrometer (ThermoFisher Scientific).

**19.1. Metabolomic data processing**

Xcalibur software (Thermo Fisher Scientific, v4.3) was used for data acquisition, and Compound Discoverer 3.2 was used for data analysis. Raw data were aligned by retention time, features were merged, and data were grouped into experimental groups. A QC mix sample was used for the identification of metabolites by annotating MS/MS spectra using mzCloud, mzLogic, and a custom mass list database with retention time. After annotation, the data were analyzed using descriptive and differential analysis. The relative levels of analytes were determined based on peak areas.

Raw data are available as a supplementary Excel file to the manuscript in the public repository.

**19.2. Plasma metabolomic data**

In the metabolomic analysis, principal component analysis (PCA) and heatmap visualizations of metabolite level changes following MI-883 treatment indicate no significant effect of MI-883 on the plasma metabolome (Supplementary Fig. 18a, b). The only notable findings were significant increases in plasma levels of isoleucine (1.638-fold, P=0.0285), 2-hydroxy-3-oxopropanoate (1.595-fold, P=0.0499), and 5-aminopentanoic acid (or 5-aminovalerate) (1.375-fold, P=0.018). No other metabolites or analytes showed significant changes in MI-883-treated mice during the proof-of-concept study.

Isoleucine, which is pro-glucogenic and ketogenic, is one of three branched-chain amino acids (BCAAs) together with leucine and valine. Mice undergoing fasting showed increased [plasma levels](https://www.sciencedirect.com/topics/biochemistry-genetics-and-molecular-biology/blood-level) of all three BCAAs which are known as fasting markers ^24^. In addition, blood levels of the BCAAs have long been connected with peripheral and hepatic insulin sensitivity mainly by activating gluconeogenesis^25^. In our proof-of-concept study, however, we observed only a significant upregulation of plasmatic isoleucine concentration (1.6-fold), but not leucine and valine (see Supplementary Fig. 18).


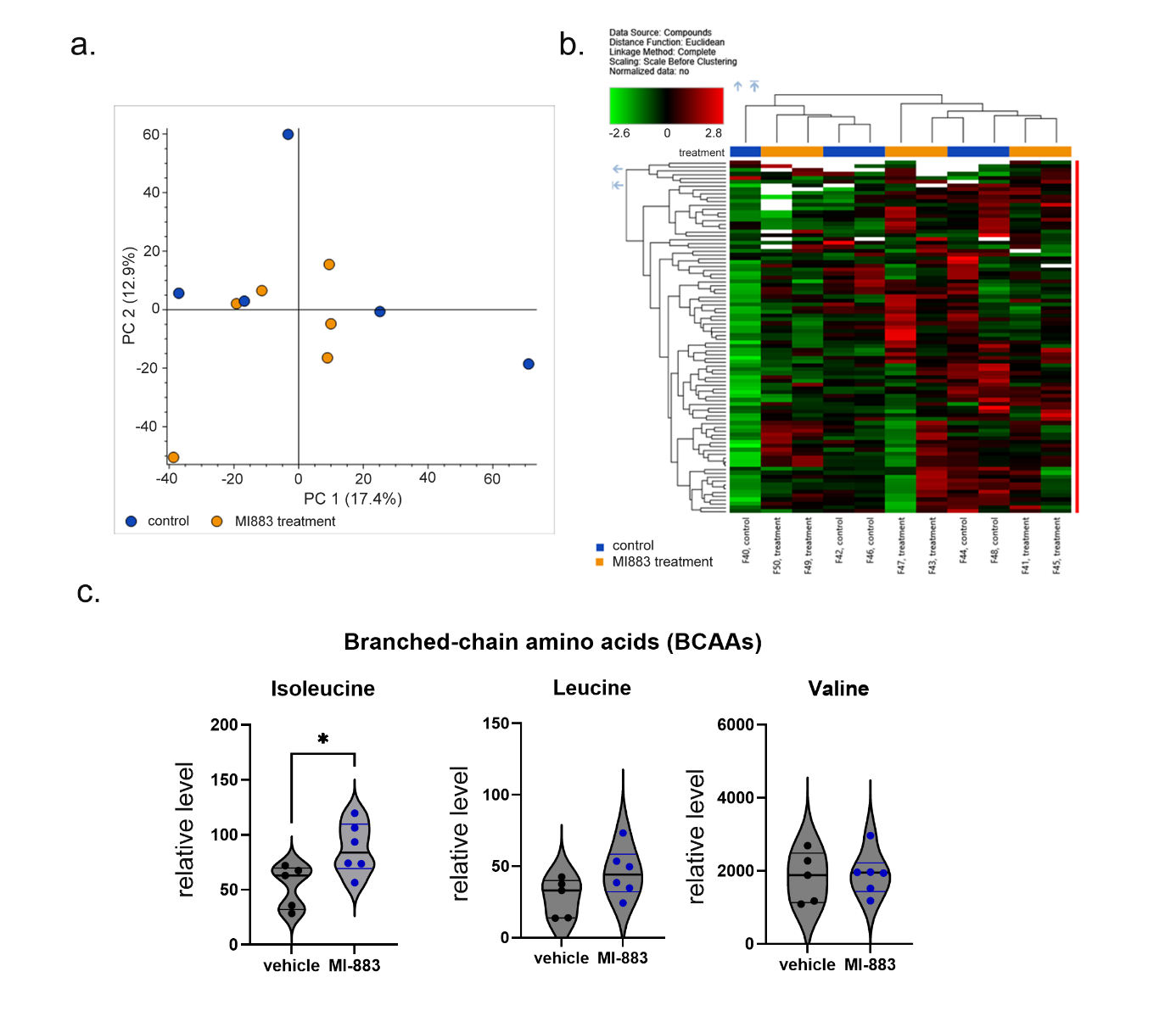


**Supplementary Figure 18.** Metabolomics of plasma samples and relative plasma levels of branched-chain amino acids (BCAAs) after treatment with MI-883 in the proof-of-concept studies. (**a**) Principal component analysis and (**b**) heatmap of metabolite concentration changes after MI-883 treatment. (**c**) Bar graph showing data for a specific metabolite, isoleucine, leucine, and valine, in the plasma of male humanized PXR/CAR/CYP3A mice treated with MI-883 in the proof-of-concept study with high-fat diet. **P* value <0.05 (Mann-Whitney U test).

The prognostic value of an increased 2-hydroxy-3-oxopropanoate (1.595-fold, P=0.0498) is not known from the literature. 2-hydroxy-3-oxopropanoate (syn. tartronate semialdehyde) is involved in ascorbate and aldarate metabolism, and glyoxylate and dicarboxylate metabolism (KEGG). The Human Metabolome Database (HMDB) indicates that tartronate semialdehyde is a potential biomarker for the consumption of some foods, which is however, irrelevant for our study.

5-Aminopentanoic acid (or 5-aminovalerate) is a lysine degradation product. Endogenous 5-aminopentanoic acid is thought to be primarily a microbial metabolite of lysine catabolism produced by the gut microbiome or oral microflora, although it can also be produced in endogenous metabolism (https://hmdb.ca/metabolites/HMDB0003355).

**20. References**

1 Mejdrova, I. *et al.* Discovery of Novel Human Constitutive Androstane Receptor Agonists with the Imidazo[1,2-a]pyridine Structure. *J Med Chem* (2023).

2 Burk, O. *et al.* Identification of approved drugs as potent inhibitors of pregnane X receptor activation with differential receptor interaction profiles. *Archives of toxicology* **92**, 1435-1451 (2018).

3 Geick, A., Eichelbaum, M. & Burk, O. Nuclear receptor response elements mediate induction of intestinal MDR1 by rifampin. *The Journal of biological chemistry* **276**, 14581-14587 (2001).

4 Mathas, M. *et al.* Evolutionary history and functional characterization of the amphibian xenosensor CAR. *Mol Endocrinol* **26**, 14-26 (2012).

5 Bitter, A. *et al.* Pregnane X receptor activation and silencing promote steatosis of human hepatic cells by distinct lipogenic mechanisms. *Archives of toxicology* **89**, 2089-2103 (2015).

6 Xu, R. X. *et al.* A structural basis for constitutive activity in the human CAR/RXRalpha heterodimer. *Molecular cell* **16**, 919-928 (2004).

7 Huber, A. D. *et al.* Mutation of a single amino acid of pregnane X receptor switches an antagonist to agonist by altering AF-2 helix positioning. *Cell Mol Life Sci* **78**, 317-335 (2021).

8 Ngan, C. H. *et al.* The structural basis of pregnane X receptor binding promiscuity. *Biochemistry* **48**, 11572-11581 (2009).

9 Schuster, D. & Langer, T. The identification of ligand features essential for PXR activation by pharmacophore modeling. *Journal of chemical information and modeling* **45**, 431-439 (2005).

10 Rashidian, A. *et al.* Discrepancy in interactions and conformational dynamics of pregnane X receptor (PXR) bound to an agonist and a novel competitive antagonist. *Comput Struct Biotechnol J* **20**, 3004-3018 (2022).

11 Teotico, D. G., Bischof, J. J., Peng, L., Kliewer, S. A. & Redinbo, M. R. Structural basis of human pregnane X receptor activation by the hops constituent colupulone. *Molecular pharmacology* **74**, 1512-1520 (2008).

12 Motta, S., Callea, L., Giani Tagliabue, S. & Bonati, L. Exploring the PXR ligand binding mechanism with advanced Molecular Dynamics methods. *Sci Rep* **8**, 16207 (2018).

13 Beekmann, K. *et al.* The effect of glucuronidation on isoflavone induced estrogen receptor (ER)alpha and ERbeta mediated coregulator interactions. *J Steroid Biochem Mol Biol* **154**, 245-253 (2015).

14 Koppen, A. *et al.* Nuclear receptor-coregulator interaction profiling identifies TRIP3 as a novel peroxisome proliferator-activated receptor gamma cofactor. *Mol Cell Proteomics* **8**, 2212-2226 (2009).

15 Carr, R. M. & Ahima, R. S. Pathophysiology of lipid droplet proteins in liver diseases. *Exp Cell Res* **340**, 187-192 (2016).

16 Lastuvkova, H. *et al.* Carvedilol impairs bile acid homeostasis in mice: implication for nonalcoholic steatohepatitis. *Toxicol Sci* **196**, 200-217 (2023).

17 Skoda, J. *et al.* Diazepam Promotes Translocation of Human Constitutive Androstane Receptor (CAR) via Direct Interaction with the Ligand-Binding Domain. *Cells* **9** (2020).

18 Pavek, P. *et al.* Gene Expression Profiling of 1alpha,25(OH)(2) D(3) Treatment in 2D/3D Human Hepatocyte Models Reveals CYP3A4 Induction but Minor Changes in Other Xenobiotic-Metabolizing Genes. *Mol Nutr Food Res* **66**, e2200070 (2022).

19 Skoda, J. *et al.* Off-target lipid metabolism disruption by the mouse constitutive androstane receptor ligand TCPOBOP in humanized mice. *Biochemical pharmacology* **197**, 114905 (2022).

20 Cohen, A. *et al.* Total serum cholesterol by isotope dilution/mass spectrometry: a candidate definitive method. *Clinical chemistry* **26**, 854-860 (1980).

21 Lenicek, M. *et al.* Improved HPLC analysis of serum 7alpha-hydroxycholest-4-en-3-one, a marker of bile acid malabsorption. *Clinical chemistry* **54**, 1087-1088 (2008).

22 Leníček, M., Vecka, M., Žížalová, K. & Vítek, L. Comparison of simple extraction procedures in liquid chromatography-mass spectrometry based determination of serum 7α-hydroxy-4-cholesten-3-one, a surrogate marker of bile acid synthesis. *Journal of Chromatography B* **1033-1034**, 317-320 (2016).

23 Jang, C., Chen, L. & Rabinowitz, J. D. Metabolomics and Isotope Tracing. *Cell* **173**, 822-837 (2018).

24 Canfield, C. A. & Bradshaw, P. C. Amino acids in the regulation of aging and aging-related diseases. *Translational Medicine of Aging* **3**, 70-89 (2019).

25 Lynch, C. J. & Adams, S. H. Branched-chain amino acids in metabolic signalling and insulin resistance. *Nat Rev Endocrinol* **10**, 723-736 (2014).
